# Evaluation of the protective efficacy of a spatial repellent to reduce malaria incidence in children in western Kenya compared to placebo: study protocol for a cluster-randomized double-blinded control trial (the AEGIS program)

**DOI:** 10.1186/s13063-022-06196-x

**Published:** 2022-04-05

**Authors:** Eric O. Ochomo, John E. Gimnig, Achuyt Bhattarai, Aaron M. Samuels, Simon Kariuki, George Okello, Bernard Abong’o, Eunice A. Ouma, Jackline Kosgei, Stephen Munga, Kiambo Njagi, Wycliffe Odongo, Fang Liu, John P. Grieco, Nicole L. Achee

**Affiliations:** 1 Kenya Medical Research Institute, Centre for Global Health Research, Kisumu, Kenya; 2 Centers for Disease Control and Prevention, Division of Parasitic Diseases and Malaria, Atlanta, GA USA; 3 National Malaria Control Program, Ministry of Health, Kenyatta National Hospital, Nairobi, Kenya; 4 Department of Applied and Computational Mathematics and Statistics, University of Notre Dame, Notre Dame, IN USA; 5 Department of Biological Sciences, Eck Institute for Global Health, University of Notre Dame, Notre Dame, IN USA

**Keywords:** Spatial repellent, Malaria, Transfluthrin, Clinical trial, Kenya

## Abstract

**Background:**

Spatial repellents are widely used for prevention of mosquito bites and evidence is building on their public health value, but their efficacy against malaria incidence has never been evaluated in Africa. To address this knowledge gap, a trial to evaluate the efficacy of Mosquito Shield™, a spatial repellent incorporating transfluthrin, was developed for implementation in Busia County, western Kenya where long-lasting insecticidal net coverage is high and baseline malaria transmission is moderate to high year-round.

**Methods:**

This trial is designed as a cluster-randomized, placebo-controlled, double-blinded clinical trial. Sixty clusters will be randomly assigned in a 1:1 ratio to receive spatial repellent or placebo. A total of 6120 children aged ≥6 months to 10 years of age will be randomly selected from the study clusters, enrolled into an active cohort (baseline, cohort 1, and cohort 2), and sampled monthly to determine time to first infection by smear microscopy. Each cohort following the implementation of the intervention will be split into two groups, one to estimate direct effect of the spatial repellent and the other to estimate degree of diversion of mosquitoes and malaria transmission to unprotected persons. Malaria incidence in each cohort will be estimated and compared (primary indicator) to determine benefit of using a spatial repellent in a high, year-round malaria transmission setting. Mosquitoes will be collected monthly using CDC light traps to determine if there are entomological correlates of spatial repellent efficacy that may be useful for the evaluation of new spatial repellents. Quarterly human landing catches will assess behavioral effects of the intervention.

**Discussion:**

Findings will serve as the first cluster-randomized controlled trial powered to detect spatial repellent efficacy to reduce malaria in sub-Saharan Africa where transmission rates are high, insecticide-treated nets are widely deployed, and mosquitoes are resistant to insecticides. Results will be submitted to the World Health Organization Vector Control Advisory Group for assessment of public health value towards an endorsement to recommend inclusion of spatial repellents in malaria control programs.

**Trial registration:**

ClinicalTrials.govNCT04766879. Registered February 23, 2021.

**Supplementary Information:**

The online version contains supplementary material available at 10.1186/s13063-022-06196-x.

## Administrative information

Note: the numbers in curly brackets in this protocol refer to SPIRIT checklist item numbers. The order of the items has been modified to group similar items (see http://www.equator-network.org/reporting-guidelines/spirit-2013-statement-defining-standard-protocol-items-for-clinical-trials/).

**Table:** 

Title {1}	Evaluation of the protective efficacy of a spatial repellent to reduce malaria incidence in children in western Kenya compared to placebo: study protocol for a cluster-randomized double-blinded control trial (the AEGIS program)
Trial registration {2a and 2b}.	ClinicalTrials.gov NCT04766879. Registered on February 23, 2021.
Protocol version {2}	Version 7, November 20, 2020
Funding {4}	The project under which the data will be gathered, “Advancing Evidence for the Global Implementation of Spatial Repellents (AEGIS),” is made possible thanks to Unitaid funding and support. Unitaid is a global health agency engaged in finding innovative solutions to prevent, diagnose, and treat diseases more quickly, effectively, and for affordable prices, in low- and middle-income countries. Unitaid’s work includes funding initiatives to address major diseases such as HIV/AIDS, malaria, and tuberculosis, as well as HIV co-infections and co-morbidities such as cervical cancer and hepatitis C, and cross-cutting areas, such as fever management. Unitaid is now applying its expertise to address challenges in advancing new therapies and diagnostics for the COVID-19 pandemic, serving as a key member of the Access to COVID Tools Accelerator. Unitaid is hosted by the World Health Organization. Additionally, SC Johnson, A Family Company (SCJ) will use internal company financial resources for the development, manufacturing, delivery, and shipment of the intervention used in the study.
Author details {5a}	EOO, SK, GO, BA, EU, JK, SM: Kenya Medical Research Institute, Centre for Global Health Research, Kisumu, Kenya JEG, AB, AMS, WO: Centers for Disease Control and Prevention, Division of Parasitic Diseases and Malaria, Atlanta, USA KN: National Malaria Control Program, Ministry of Health, PO Box 19982 Kenyatta National Hospital, Nairobi 00202, Kenya JPG, NLA, FL: University of Notre Dame, Notre Dame, IN 46556, USA
Name and contact information for the trial sponsor {5b}	Dr. John P. Grieco Lead Principal Investigator, Advancing Spatial Repellents for Vector-Borne Disease Control Eck Institute for Global Health University of Notre Dame 243 Galvin Life Science Notre Dame, IN 46556 jgrieco@nd.edu 574.631.7572
Role of sponsor {5c}	As study sponsor, UND participated in study design, management, analysis, data interpretation, and manuscript development As funder, Unitaid had no role in the design of the study and collection, analysis, and interpretation of data and in the writing of the manuscript.

## Introduction

## Background and rationale {6a}

Despite intensive scale up of insecticide-treated nets (ITNs) and effective anti-malarials, malaria remains one of the primary causes of morbidity and mortality in the region [1]. Malaria transmission and burden in areas of western Kenya have been reported to be among the highest in the world. After a period of decline, malaria burden has remained stable over the last few years and may be increasing in some areas. New prevention and control tools are needed to further reduce malaria transmission and accelerate progress towards elimination and eventual eradication [2], which includes addressing the threat of insecticide resistance and outdoor biting vectors [3].

Spatial repellents (SRs) are a promising new vector control paradigm that could add to the existing armamentarium for malaria prevention [4–7], and the World Health Organization (WHO) has recommended methods to evaluate the efficacy of new SR products [8]. SRs are products that contain chemicals that reduce human-vector contact by eliciting a range of behaviors in insect vectors [9], including movement away from chemical stimuli [10], interference with host detection, attraction inhibition, and/or reduced feeding response [11] providing (1) protection against daytime, early-evening biting; (2) protection in enclosed/semi-enclosed and peri-domestic spaces; (3) a range of formulation options to fit context-specific application requirements thereby facilitating health systems strengthening; and (4) increased coverage of vector control over traditional methods. In addition, SR product active ingredients (AIs) have demonstrated increased attraction to oviposition cues [12] that could intervene in the vector life-cycle or enhance combination interventions (i.e., push-pull) [13] and have demonstrated effect on mosquito fecundity [14] and against insecticide-resistant vector species linked to malaria transmission [15]. Currently, the majority of commercial SR products utilize either low concentrations of short-duration United States Environmental Protection Agency (USEPA) [16] registered synthetic pyrethroids (pyrethrin, metofluthrin, and more recently transfluthrin) or botanical-based compounds [7, 17].

Existing malaria vector control guidelines do not recommend the use of SRs for personal protection [18]. However, various SR products have been shown to reduce mosquito biting [19–25] and, in epidemiological studies conducted in Indonesia [26], China [27], and Peru [28] have been shown to reduce pathogen transmission in human populations. In Indonesia, using a transfluthrin-based SR product, Shield with a 2-week duration of protection, a 27.7% reduction in time to first-event and 31.3% in overall infections was demonstrated, but outcomes were not statistically significant due to low incidence in some clusters at baseline, undermining the power to detect a protective effect [26]. Additionally, the study demonstrated a 16.4% and 11.3% reduction in anopheline attack rates indoors and outdoors of households (HHs), respectively. In the Peru trial, the same SR intervention was used, Shield, which significantly reduced arboviral infections by 34.1% with an associated reduction in *Aedes aegypti* abundance and blood-fed capture rates by 28.6% and 12.4%, respectively [28].

The Kenya trial described here will evaluate the efficacy of a next-generation formulation of the SR product used in the Indonesia and Peru trials, Mosquito Shield™, with a duration of protection up to 4 weeks. The choice to use the Mosquito Shield^TM^ product was based on this product containing the same active ingredient and design (i.e., passive emanator) as was used in clinical trials dating back to 2013 which demonstrated impact to reduce malaria and arbovirus infections [28, 29]. Thus, preliminary public health value data exists for this “first-in-kind” prototype for the SR class. The study also been designed to demonstrate whether deployment of SRs will or will not result in increased malaria transmission and disease among geographically proximal non-users.

This evaluation will serve as a proof-of-principle of SR product protective effect for sub-Saharan Africa. Trial results will be submitted to the WHO Vector Control Advisory Group (VCAG) to contribute to the body of evidence regarding the impact of SRs in reducing human malaria infections in settings where underlying transmission rates are high at baseline, ITNs are widely deployed, and/or where mosquito vectors bite outdoors. The outcomes of this study will inform policy recommendations for SRs as a means to further reduce malaria transmission.

### Objectives {7}

The study’s primary objective is to demonstrate and quantify the protective efficacy (PE) of the Mosquito Shield™, a transfluthrin-based SR product, in reducing the incidence of malaria infection in humans.

Secondary objectives will address issues related to the optimization and application of SR products for public health and confirm the range of contexts within which SR PE can be achieved. Secondary objectives are:

1. To estimate the total number of infections averted due the SR intervention by comparing the number of infections per person per year in intervention and control arms over the 2-year follow-up period;
2. To investigate whether the SR intervention induces diversion of mosquitoes from within intervention areas to locally unprotected individuals thereby having a differential impact on infection incidence among protected and locally unprotected individuals;
3. To measure the impact of the SR intervention on entomological correlates of transmission (e.g., vector densities, mosquito infection, host seeking/biting and parity rates) to set benchmark thresholds and streamline future intervention trials with other SR products by measuring those endpoints correlated to PE.

### Trial design {8}

An outline of the trial design and sample size is summarized in Table 1. This study is a cluster-randomized controlled trial (cRCT) with 30 clusters per treatment arm (SR and placebo), consisting of 4 months of baseline data collection and after baseline, two independent cohorts enrolled (cohort 1 and cohort 2) and each followed for 12 months for a total of a 24-month follow-up with intervention.

**Table 1:** Outline of the cluster design and sample size for the SR cRCT in Busia County, Kenya

Cluster design	Baseline cohort	Intervention cohorts		
	4-month follow-up (*n* = 2040)	Time of intervention deployment	12-month follow-up (cohort 1) (*n* = 2040)	12-month follow-up (cohort 2) (*n* = 2040)
**Core area** 60 clusters total	20 subjects per cluster 1200 subjects total	Year 1	SR (30 clusters), Placebo (30 clusters) 14 subjects per cluster 840 subjects total	n/a
		Year 2	n/a	SR (30 clusters), Placebo (30 clusters) 14 subjects per cluster 840 subjects total
**Buffer zone** 60 clusters total	14 subjects per cluster 840 subjects total	Year 1	SR (30 clusters), Placebo (30 clusters) 20 subjects per cluster 1200 subjects total	n/a
		Year 2	n/a	SR (30 clusters), Placebo (30 clusters) 20 subjects per cluster 1200 subjects total

For the evaluation of the primary epidemiological objective (PE), a total of 28 subjects (HHs) will be recruited from each core area of a cluster or equivalently 14 subjects (HHs) per cohort per cluster (factoring in a 35% loss-to-follow-up (LTFU) rate). For the evaluation of the secondary epidemiological objective on SR diversionary effect, a total of 40 subjects (HHs) or equivalently 20 subjects (HHs) per cohort per cluster will be recruited from each buffer zone (factoring in a 35% LTFU rate). At least one child aged ≥ 6 months old to 9 years and 11 months old from each HH will be recruited for biweekly (every 2 weeks) malaria check-ups during the follow-up period with intervention.

Twenty clusters (10 SR, 10 placebo) will be randomly selected to estimate the impact of the SR on entomological measures of malaria transmission. These clusters will remain fixed throughout the study. Within each of the 20 clusters, light trap collections will be conducted monthly in 10 randomly selected HHs within the core area of each sentinel cluster to assess the impact of SRs on the density of *Anopheles* mosquitoes indoors. On the same night, light trap collections will also be conducted in 15 randomly selected HHs in the buffer zone of the same cluster to estimate the diversionary effect of the SR. Human landing catches (HLC) will be performed indoors and outdoors of 48 HHs in a subset of 12 (6 SR intervention, 6 placebo) of the 20 clusters selected for entomology evaluation to determine the effect of SR on the host seeking behavior of mosquitoes. The 12 clusters will remain fixed throughout the study. Sampling will be performed in four houses (randomly selected) in each cluster for two nights once every quarter (3 months). The same houses will be sampled each quarter.

**Fig. 1:**
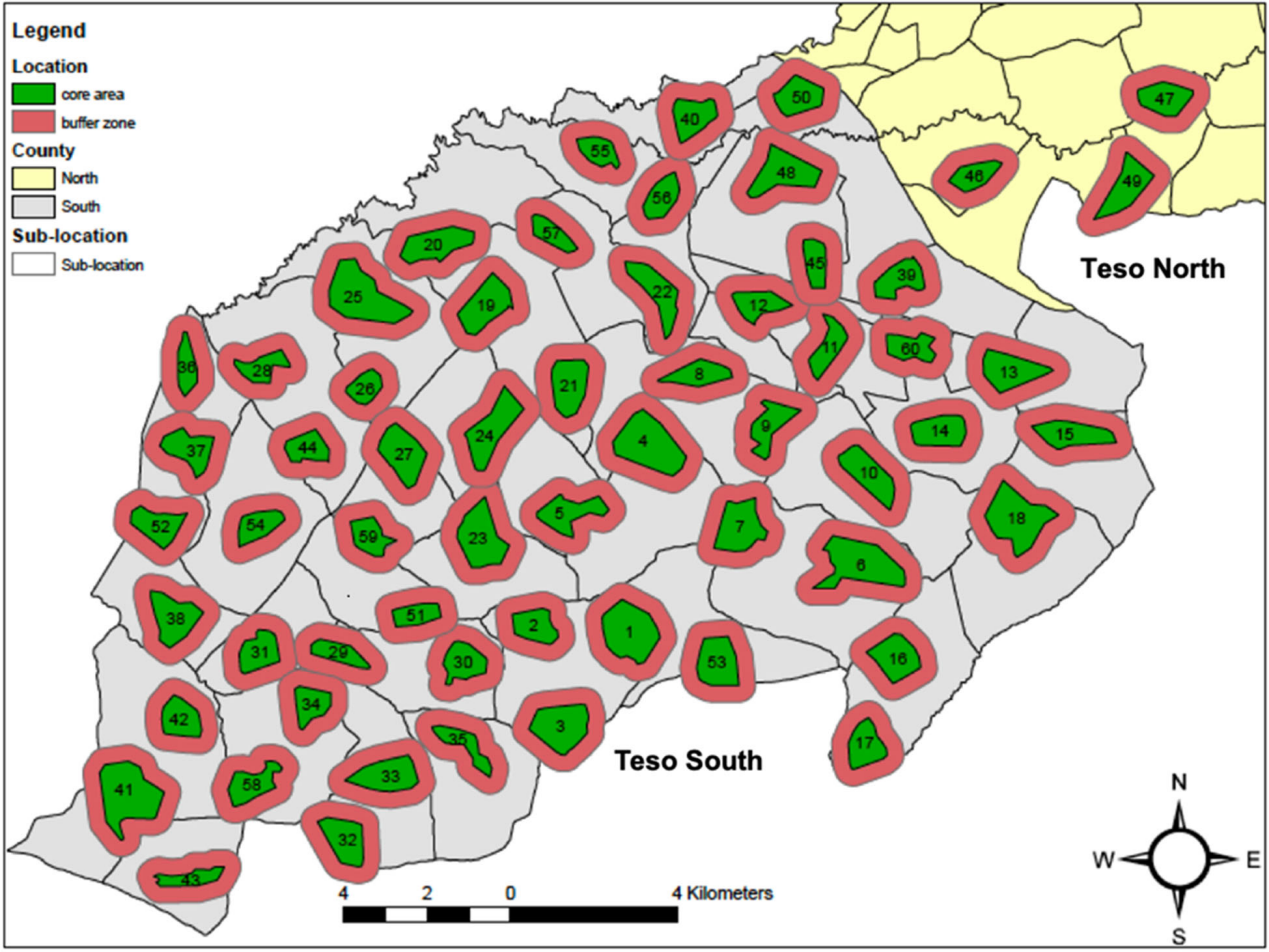
Location of 60 study clusters in Teso North and Teso South Districts, Busia County, western Kenya. Each cluster consists of a core area (discrete village) and a buffer zone extending 300–500 m outside the core area in which diversionary effects of the SR intervention on epidemiological and entomological endpoints will be measured

Each cluster will consist of a core area, which corresponds to an existing village, and a buffer zone which includes HHs outside but within 300–500 m of the core area (Fig. 1). Based on studies evaluating mass effect of ITNs [30], endpoints measured in the buffer zone in our trial will be used to quantify induced diversion by the SR intervention on mosquitoes from within cluster core areas to locally unprotected individuals in the buffer zones thereby potentially having a differential impact on malaria infection incidence among protected and locally unprotected individuals.

## Methods: participants, interventions, and outcomes

### Study setting {9}

The study will be conducted in Busia County, which is located on the border with Uganda, approximately 100 km northwest of Kisumu City. Busia County has a population of 743,946 which is made up of primarily the Luo, Luhya, and Teso ethnic groups. The population of the area identified for this study is predominantly of the Teso ethnic group. The population lives in scattered homesteads and survives primarily as subsistence farmers. Malaria transmission is high and perennial with seasonal peaks in June–July and October–November which follow rainfall patterns. The long rains occur from late March to early June and the short rains occur around October through November. The primary mosquito vectors observed in the area include *An. gambiae s.s.*, *An. arabiensis*, and *An. funestus* [31].

In the 2015 Malaria Indicator Survey, malaria prevalence among children aged 6 months to 14 years in the Lake endemic region was 42.4% by rapid diagnostic test (RDT) and 26.7% by microscopy [32]. A study conducted in Busia County in 2013–2014 found the incidence of malaria infections ranged between 2.5 and 4.1 per person per year [33]. ITN coverage is high, with 88.2% of HHs owning at least one ITN and 68.4% of persons reporting sleeping under a net the previous night [32]. Due to the continued high burden of malaria despite high coverage of ITNs and the presence of KEMRI/CDC malaria research in the region, this site was selected to evaluate the efficacy of the Mosquito Shield™ intervention.

### Eligibility criteria {10}

The inclusion and exclusion criteria are listed in Table 2. All children aged ≥ 6 months to 10 years of age who report sleeping in selected study clusters > 90% of the nights of each month and do not have any plans to travel outside the study area (for more than 5 consecutive weeks) will be eligible for inclusion in the study. Children aged 5 to < 10 years have the highest malaria prevalence in this area, and so it was deemed important to include them to estimate the potential benefit of SR on this population. This will allow us to assess the impact on different age groups and infer the added benefit of the Mosquito Shield™ on top of long-lasting insecticidal nets (LLINs), for which coverage is lower among older aged children. Children who have a measured hemoglobin at baseline of ≤5 mg/dL or < 6 mg/dL with signs of clinical decompensation will be excluded from the study. Persons who are participating in another clinical trial investigating a drug, vaccine, medical device, or procedure will also be excluded from the study. The written consent of the parent or legal guardian of each child is required for inclusion in the study.

**Table 2:** Inclusion and exclusion study criteria

Inclusion criteria	Exclusion criteria
Children aged 6 months to < 10 years	Children < 6 months or ≥ 10 years
Hb > 5 mg/dl	Hb ≤ 5 mg/dL, or Hb < 6 mg/dL with signs of clinical decompensation
Sleeps in cluster > 90% of nights during any given month	Sleeps in cluster < 90% of nights during any given month
No plans for extended travel (> 1 month) outside of home during study	Plans for extended travel (> 1 month) outside of home during study
Not participating in another clinical trial investigating a vaccine, drug, medical device, or a medical procedure during the trial	Participating or planned participation in another clinical trial investigating a vaccine, drug, medical device, or a medical procedure during the trial
Provision of informed consent form (ICF) signed by the parent(s) or guardian	No provision of ICF signed by the parent(s) or guardian
Children not on regular malaria prophylaxis***	Children on regular malaria prophylaxis***
Willingness to take Artemether-Lumefantrine (AL) and no history of hypersensitivity to AL	Unwillingness or refusal to take AL and history of AL hypersensitivity

*** Malaria prophylaxis medicines: Mefloquine, Atavaquone/Proguanil (Malarone), Doxycycline, Sulfadoxine-Pyrimethamine (Fansidar), Amodiaquine, and Co-trimoxazole (Septrin)

### Who will take informed consent? {26a}

Informed consent will occur for three major study activities: (1) malaria incidence follow-up, (2) product application inside homes, and (3) mosquito collections. The study will be explained in local dialects (Teso and Kiswahili), and time allocated for questions to be answered. Once all concerns are addressed, ICFs will be signed.

For consent to malaria incidence follow-up, the study will first be verbally explained to the head of compounds and HHs during which their permission will be sought to list and randomize eligible children in the compound. A single child from the master randomization list will be recruited, and an ICF will be read to the parent/guardian of the child to undergo screening for enrolment. An attempt to obtain written, informed consent from both parents of the child will be made, but consent from only one parent will be required for participation. Parent(s)/guardian(s) who cannot sign their names will provide a thumb print on the ICF for documentation of willingness to participate, and a witness not associated with the study will sign the ICF indicating the form was read, and that participation and thumb-printing were given willingly without coercion. Time will be granted to those parent(s)/guardian(s) who would wish to make consultations with their family members before signing. It will be stressed to all parents/guardians approached that their children’s entry into the study is voluntary and they may withdraw from the study at any time for any reason without any penalty. In HHs with multiple children meeting the study inclusion criteria, only one eligible child will be invited to participate. Consented subjects will be assigned a subject identity code on enrolment. Consented participants will be screened for inclusion/exclusion criteria.

For product application, all HHs within the selected clusters will be eligible to receive intervention. Informed consent from heads of HH, or spouse, will be sought for application of product in the home. Heads of HH will also be consented for mosquito light trap and HLC collections inside and/or outdoors of their homes. HH with child subjects enrolled for malaria incidence follow-up will be excluded from HLC collections. Adult males residing in consenting homes will be recruited to serve as mosquito collectors. The collectors will be asked to stay up throughout the night collecting mosquitoes that land on their exposed legs. An ICF will be obtained from the collectors.

All ICFs will include informing participants to report to a study clinic if experiencing an adverse event (AE) to be assessed and receive essential, point of care treatment free of charge whether or not the event is subsequently determined to be study related. They will only receive enough treatment to ensure they are not disadvantaged, clinically or financially, by going first to the study clinician instead of regular Ministry of Health (MOH) staff. They will not, however, receive free, comprehensive treatment for any illness found to be unrelated to study procedures (product, medicines). The ICF will also inform participants if anyone in their house becomes severely ill, they should go immediately to the nearest health facility.

Copies of informed consent documents will be made available upon request to the AEGIS Program Manager (aegis@nd.edu).

### Additional consent provisions for collection and use of participant data and biological specimens {26b}

This is not applicable—participant data and biological specimens will not be used in ancillary studies.

## Interventions

### Explanation for the choice of comparators {6b}

According to the WHO VCAG’s guidelines for vector control field trial design, studies should always have a control group from which data collection occurs contemporaneously with data collection from the intervention group [34]. Our trial design includes a placebo product of matched design to the Mosquito Shield™ but with inert ingredients only. The use of a placebo is generally acceptable when a placebo is compared against an intervention in combination with standard treatment [35]. Our study design will not withhold standard-of-care for clinical management of malaria nor standard-of-care vector control interventions (e.g., LLINs, indoor residual spraying (IRS)) in either the SR or placebo arm, but instead, these will be monitored and recorded throughout the trial. This approach is aligned with WHO VCAG guidance that the control group must receive care reflecting the standard best practice interventions like routine vector control measures, such as LLINs distributed through mass campaigns, and routine health systems such as antenatal clinics or Expanded Programme on Immunization (EPI) visits. Study participants will also be encouraged to continue use of LLINs and not instructed to avoid alternative vector control tools (e.g., coils, topicals, aerosol sprays, repellents).

### Intervention description {11a}

The SR product used for the study will be a new formulation of transfluthrin, Mosquito Shield^TM^, a passive emanator that releases active ingredient over a period of up to 4 weeks. Transfluthrin is widely used in mosquito coils and other HH pest control products worldwide. The emanator consists of a pre-treated medium with a standard amount of transfluthrin that will be present throughout the treated space continuously based on a standardized 4-week replacement schedule. Products will be positioned along interior walls (approximately 2–3 m above ground) according to manufacturer specifications of 2 units per 9 m^2^. More than one emanator may be applied in a HH depending on the size of the house. A placebo product of matched design with inert ingredients will be applied similarly. Each product (SR and placebo) will have a unique code associated with an individual cluster, which will be recorded at the time of installation and replacement. Both participants and study staff will be blinded as to whether the product contains transfluthrin or is a placebo. The head of HH may be engaged about their perceptions and acceptability of the product after the product has been deployed.

### Criteria for discontinuing or modifying allocated interventions {11b}

If a participant chooses to discontinue participation in the study, study staff will respect the decision without penalty. Study staff may terminate subject participation at any time during the trial, as needed, if a subject no longer meets the inclusion criteria and/or based on AE and/or SAE clinical assessment.

### Strategies to improve adherence to interventions {11c}

In order to promote adherence to intervention, study staff will be employed to ensure the appropriate placement, quantity, and replacement schedule of products in HHs. Additionally, study staff will perform periodic, unannounced spot checks in HHs to confirm products are properly installed. If during scheduled product replacement a product has been found to have been moved after application, study teams will record for use in qualitative assessment of HH compliance. If needed, study staff will re-engage with heads of HHs to discuss importance of maintaining original product placement. Overall product coverage will be estimated based on total HHs recorded having product volume at time replacement at required levels according to manufacturer specifications (2 units/9 m^2^).

### Relevant concomitant care permitted or prohibited during the trial {11d}

Our study design will not withhold standard-of-care for clinical management of malaria nor standard-of-care vector control interventions (e.g., LLINs, IRS) in either the SR or placebo arm, but instead, these will be monitored and recorded throughout the trial. All children enrolled in the cohort will be provided a new LLIN. Study participants will be encouraged to continue use of LLINs and not instructed to avoid alternative vector control tools (e.g., coils, topicals, aerosol sprays, repellents). This will allow us to estimate the effect of the SR assuming all other measures are still occurring for malaria prevention, essentially providing insight on an additive benefit above that provided by currently recommended WHO malaria preventive measures. At baseline, children enrolled into the cohorts will be provided a treatment dose of AL free of charge according to Kenya national treatment guidelines to clear any prepatent or patent malaria parasites. Lastly, subjects will be provided treatment for malaria infection throughout the follow-up period.

### Provisions for post-trial care {30}

This is not applicable—the study will not provide post-trial care.

### Outcomes {12}

The primary outcome measure of this cRCT will be the first-time malaria incidence rate as measured by microscopy in children aged ≥ 6 months to 10 years.

Secondary outcome measures include:

1. Overall new *Plasmodium falciparum* malaria infections, as measured by microscopy;
2. The first-time and overall *P. falciparum* malaria infections in buffer zones, as measured by microscopy;
3. The first-time and overall *P. falciparum* malaria infections by two age groups (≤ 59 months old; 5 years old to 9 years and 11 months old), as measured by microscopy;
4. Anopheline-human contact (indoor and outdoor) using human biting rate (HBR) as an indicator for all anophelines and by anopheline species, as measured by HLC during 12-h intervals on a quarterly basis;
5. Anopheline survival and population age structure using parity rate as an indicator for all anopheline and by anopheline species, as measured by mosquito ovarian dissections from a sub-sample of anophelines collected during HLC procedures;
6. Anopheline infectivity using sporozoite rate as an indicator for all anopheline and by anopheline species, as measured by laboratory detection of sporozoites in mosquito heads and thoraces from a sub-sample of anophelines collected during HLC and/or CDC light trap collections;
7. Entomological inoculation rate (EIR) as an indicator of transmission intensity for all anophelines and by anopheline species, as measured by calculating the number of sporozoite-infected anopheline mosquitoes captured per person from CDC light trap and/or HLC collections;
8. CDC light trap indoor density for all anophelines and by anopheline species, as measured by light trap collections during 12-h intervals on a monthly basis;
9. Insecticide resistance, as measured by WHO filter paper test and CDC bottle assays during baseline and intervention phase.
10. Adverse events (AEs) and serious adverse events (SAEs), as measured by solicited and unsolicited reports during baseline and intervention phase. Mean, minimum, and maximum frequency and percentage of AEs and SAEs across clusters among enrolled subjects will be summarized by treatment arm.

### Participant timeline {13}

The participant timeline is shown below.

**Table:** 

	Study period			
	Baseline	Intervention		Close-out
**Timepoint**	(Mar 2021–Jul 2021)	(Aug 2021–Jul 2022)	(Aug 2022–Jul 2023)	(Aug 2023–Jan 2024)
**Baseline**				
**Informed consent**	X			
**Screening**	X			
**Follow-up**	X			
**Intervention - cohort 1**				
**Allocation**		X		
**Informed consent**		X		
**Screening**		X		
**Follow-up**		X		
**Intervention - cohort 2**				
**Allocation**			X	
**Informed consent**			X	
**Screening**			X	
**Follow-up**			X	
**Assessments**				
**Baseline interim analysis**	X			
**Baseline final analysis**		X		
**Interim analysis (with intervention)**		X		
**Final analysis (with intervention)**				X

### Sample size {14}

A summary overview of sample size estimates is provided in Table 1.

Assumptions used to calculate the sample sizes below will be evaluated during the baseline phase and may lead to an adjustment in the overall sample size required for the intervention phase of the main trial. Since the adjustment will only utilize the estimated baseline incidence and coefficient of variance (CV) from the baseline without any intervention or randomization information, the type I error rate will not be inflated.

#### Primary hypothesis on first-time malaria infection

The sample size determination on the required number of subjects (HHs) per cluster for testing the primary hypothesis is based on the hazard rate comparison in the proportional hazards regression model [36, 37]. With the following specifications: 1-sided type I error rate = 5%, true PE = 30%, a between-cluster CV of hazard rate = 44% (based on historical data collected from Kenya), one interim analysis for efficacy and non-binding futility with the O’Brien-Fleming error spending function when 50% of events have been collected then 1055 first-time malaria events will need to be observed to reach 85% power in testing the primary hypothesis on PE.

With a baseline first-time malaria infection hazard rate of 3.0 per person-year, 30 clusters per treatment arm, with 20 subjects (HHs) per cluster are expected to yield 1055 independent first-time malaria events within a 12-month follow-up period per cohort post randomization to yield 85% power. If, by the end of the 2-year study, 1055 independent malaria events are not reached, the study may extend until 1055 events are collected without inflating the type I error rate in the testing of the primary hypothesis. Factoring in a LTFU rate at 35%, the required sample size is 28 subjects (HHs) per cluster, which will be split in half between the two sequential cohorts with 14 subjects (HHs) per cohort per cluster.

#### Secondary hypothesis on overall malaria infection

The sample size calculated to yield 85% power for establishing the primary hypothesis on first-time infection PE also leads to at least 85% power when it comes to the testing of the secondary hypothesis on the overall malaria infection. This is because that the baseline overall malaria incidence rate is likely to be no lower than 3.0 per person-year (the first-time incidence rate), and there is no interim analysis on the second hypothesis.

#### Quantification of diversionary effect

Since there is no formal hypothesis on the SR-diversion objective, we focus on determining a practically feasible sample size that will give a relatively high precision for the estimated PE of SR in the cluster buffer zone on the malaria incidence rate. With a 44% between-cluster CV, and a baseline incidence rate of 3.0 per person-year, 30 clusters per arm with 28 subjects (HHs) per cluster leads to a half width of 0.220 for the 90% confidence interval on the log scale, or the ratio between the upper bound of the 90% confidence interval versus the point estimate is 1.245 for a hazard ratio estimate between SR and placebo buffer zone subjects (HHs). Factoring in a 35% LTFU rate, the required sample size is 40 subjects (HHs) in the buffer zone per cluster. The total subjects (HHs) will be split in half between two sequential cohorts (cohort 1 and cohort 2), with 20 subjects (HHs) per cohort per cluster.

### Recruitment {15}

Information will be distributed through Community Interviewers (CIs) and community health workers, and other study staff, targeting parent(s)/guardian(s) of potential participants in the community. Fliers or posters will be developed and information will be provided through the following means: Community meetings in facilitation with local Chiefs Barazas, church meetings where recruitment scripts will be read and fliers handed to the parent(s)/guardian(s) of potential participants and discussions with church members; attendance at established local community advisory board (CAB) and opinion leaders’ meetings, women groups, and men groups.

After community entry, a list of all chief barazas, CAB meetings, Community Health Volunteers (CHVs), and community gatherings for a particular month will be prepared. The component leads, in consultation with the study coordinator and community interviewers, will organize to attend the meetings to share information about the study and plan a schedule for recruitment. If no meetings are planned during specific month when recruitment is ongoing, the study coordinator will organize meetings as needed. The recruitment team that includes the study clinician, community interviewers, and CHVs will set up one or several tents in strategic places and meet parents of potential participants. Should the community offer to use rooms in a school or church, especially during the rainy season, this will be accepted, as this will be mainly for sensitization activities. The study mobile field sites will be set up in 1 to 4 sub-locations at a given time with the guidance of the CHVs and all interested parents/legally acceptable representatives with potential participants in that area will be invited for information about the study.

Within each cluster, individual compounds will be enumerated and then randomly selected for inclusion in each cohort (baseline, cohort 1, cohort 2). Parents with children aged ≥ 6 months to 9 years 11 months will be eligible for consenting into participation in the SR study. Community interviewers will also trace pre-selected compounds where they will obtain verbal consent from head of HHs to collect baseline information which includes listing all the children between ≥ 6 months and 9 years 11 months in the compound. This master list will be used to randomly select one child per HH for subject recruitment. Compounds selected for inclusion in the baseline cohort will be eligible for inclusion in cohorts 1 or 2 after implementation of the intervention. However, those children enrolled in cohort 1 after implementation of the SR will not be eligible for enrolment in cohort 2 for the second year of follow-up.

## Assignment of interventions: Allocation

### Sequence generation {16a}

For the baseline cohort, recruitment of participants for enrolment was based on random selection of HHs using census mapping of the study area. Random allocation of clusters into treatment assignment will be performed during baseline following interim analysis of incidence and prior to cohort 1 participant enrolment. Baseline estimates of incidence will be used to guide decisions on cluster stratification. A total of 60 clusters (30 per treatment arm) will be randomly allocated to receive either the SR intervention or placebo treatment. The cluster allocation sequence will be generated by the external statistician serving on the Data Safety Monitoring Board (DSMB) using a random number generator (https://www.random.org).

### Concealment mechanism {16b}

Investigators, study biostatistician, staff, and study participants will be blinded to cluster, thus HH treatment allocation. The SR intervention and placebo will have identical design and packaging and will be deployed in houses by study personnel using a blinded coding scheme. The study biostatistician will remain blinded throughout the trial, but will conduct an unblinded analysis following database lock upon completion of all data entry and resolution of standing data queries at the end of the study.

### Implementation {16c}

The distribution of the intervention will occur after the completion of baseline phase analyses and verification of underlying assumptions on study power (incidence, CV).

All consented HHs located within cluster core areas, including those that do not have a child subject enrolled in an incidence cohort, will have product placed inside their homes at the manufacturer’s recommended application rate of 2 units per 9 m^2^ floor area. Trained study teams will be responsible for managing product implementation including initial deployment of product, subsequent removal, and replacement at 4-week intervals.

## Assignment of interventions: Blinding

### Who will be blinded {17a}

All participants, investigators, and study staff will be blinded to cluster, thus HH, allocation throughout the duration of the trial.

### Procedure for unblinding if needed {17b}

#### Non-emergency unblinding of a single participant

If, because of an AE which might be related to the SR product, and non-emergency unblinding of an individual participant is considered, unblinding will follow recommendations outlined in pre-specified standard operating procedures for non-emergency unblinding. The site clinician will inform the Site PI of the AE under consideration, and the Site Principal Investigator (PI) will contact the University of Notre Dame (UND) Lead PI and the medical monitor on the DSMB to discuss the case and obtain agreement that the participant, thus HH allocation, should be unblinded in a non-emergency manner. If unblinding is agreed upon, the sealed (digital password protected) intervention assignment will be with the Site PI but only opened by a pre-designated person external to the study (i.e., administrator) so as to maintain the Site PI’s and study staff blinding to cluster assignments. Documentation of the unblinding will be performed with a subsequent follow-up memo to the UND Lead PI, and DSMB. Reporting of non-emergency unblinding due to an AE will be conducted as prescribed by corresponding institutional review boards (IRB) by the Site PI, UND Lead PI, or designee. The possible effect of unblinding on the planned study data analysis will be determined by the Site PI or designee.

#### Emergency unblinding

Emergency unblinding will be considered in instances of a suspected unexpected SAE to the study product or procedures (malaria treatment, mosquito collection) as judged by a site physician following recommendations outlined in pre-specified standard operating procedures for emergency unblinding. The first alert will be raised by a study physician within 24 h of becoming aware of the SAE in an expedited report to the Site PI, UND Lead PI, and DSMB. Documentation of the unblinding, reporting to IRBs, and possible effects of unblinding on the planned study data analysis will follow similarly as described above.

## Data collection and management

### Plans for assessment and collection of outcomes {18a}

#### Mapping of the study area and baseline measurements

Prior to enrolment, all structures (human dwellings) in the study area will be mapped using GPS coordinates and assigned a unique identification number. A baseline questionnaire will be administered to measure HH and entomological characteristics. Profiles of enrolled HHs that could potentially confound effect on mosquitoes, thus malaria transmission, will be generated. This includes house construction materials, socioeconomic status, number of inhabitants and their age, current method(s) used to prevent mosquito bites (including ITNs), and presence of domesticated animals, density, and location. A cluster will be defined as a village which will serve as the core area where the intervention is implemented plus a buffer zone extending 300–500 m beyond the core area which will be used to estimate the diversionary effect of the intervention on epidemiological and entomological endpoints (Fig. 1) [30]. Since the buffer zones will extend into neighboring villages, an anticipated 100–150 villages will be mapped to account for buffer zones. Core areas (villages) plus their buffer zones will be identified using mapping software (ArcGIS/QGIS) based on the feasibility of identifying 60 villages plus their surrounding buffer zones.

#### Description of study clusters

The unit of randomization for the intervention will be a cluster. A total of 60 clusters will be delineated for the trial (Fig. 1). A cluster is defined as a core area, where the SR or placebo will be implemented, plus an untreated buffer zone where diversionary effects of the Mosquito Shield™ will be measured. The core areas of each cluster will be discrete villages. In the Health and Demographic Surveillance System maintained by KEMRI and CDC in neighboring Siaya County, the average village has 103 compounds in an area of 1.75 km^2^. Compounds are defined as family units with one or more structures plus surrounding farmland. A typical family compound has 5.5 HH members. Therefore, an average population size for a village in this area is estimated to be 567 inhabitants. Pre-trial census data will be used to delineate cluster boundaries based on number of HHs and inhabitant age groups such that each cluster will have at least one anticipated child meeting the inclusion criteria for assurances of sample size requirements.

For the buffer zones, mapping will extend approximately 300–500 m from the border of each cluster core area. Assuming core areas are approximately round with an area of 1.75 km^2^, this will result in a radius of 0.75 km. Extending a buffer zone 300 m around the core area will result in a radius of 1.05 km, and extending a buffer zone 500 m will result in a radius of 1.25 km. Estimating the total area including the buffer zones and then subtracting out the area of the core area results in areas of 1.7 km^2^ and 3.1 km^2^ for buffer zones of 300 m and 500 m respectively. Typically, HHs are randomly distributed in the landscape of western Kenya and it is therefore expected that a 300 m buffer zone of 1.7 km^2^ should have a similar number of HHs as the core areas which are estimated to have an area of 1.75 km^2^. Final determination of the buffer zones will be based on mapping and the actual number of HHs required to meet the sample size of 40 HHs per cluster for the analysis of SR diversionary effects. Because the buffer zones will extend into neighboring villages, we will map approximately 100–150 villages and then select villages as core areas based on feasibility of including a 300–500 m buffer around 60 of them.

#### Enrolment of the cohort

Cohorts of children aged ≥ 6 months to 9 years 11 months will be enrolled from randomly selected compounds using a master list generated during baseline mapping to achieve the desired sample size (see “Recruitment {15}”). HHs within each cluster will be identified by simple random sampling using a statistical software package such as SAS, STATA, or R. Selected HHs will be visited and all eligible children in the HH invited to participate in the study. The children will be enumerated on a tablet and a randomization program run to pick one who will be invited to participate in the study. The study will be explained and consent obtained from the child’s parent/guardian before enrolling the child in the study.

At enrolment, the age and sex of the child will be recorded and the parent/guardian will be asked about the use of ITNs and anti-malarial drugs. A fingerstick blood sample will be taken for a malaria RDT, a blood smear, and a measurement of hemoglobin. At this time, cohort participants will be presumptively cleared of parasites with a treatment dose of AL unless they have recently been treated (within the last 2 weeks) in which case, their microscopy results will be fast-tracked to confirm clearance and if not cleared, another dose of AL will be given. Children will be treated according to Rapid Diagnostic Test (RDT) result throughout the follow-up period unless the microscopy result is positive following a negative RDT or where an RDT was not done.

All enrolled children will be provided a LLIN. This will allow us to measure the added benefit of SR product above that provided by current WHO recommended malaria preventive measures. In addition, children enrolled into each baseline, cohort 1, and cohort 2 will be provided a treatment dose of AL free of charge to clear any prepatent or patent malaria parasites.

#### Cohort follow-up

Children enrolled in the baseline cohort will be followed for a 4-month period, prior to the distribution of the study products. To avoid substantial LTFU due to study fatigue, two independent cohorts (cohort 1 and cohort 2) will be recruited and followed with intervention each for 12 months (see Trial Design {8}); therefore, the total period of follow-up will be 28 months; 4-month baseline phase and 24 months after deployment of the intervention. Cohort 1 will end 12 months after the SR is deployed. Cohort 2 will be recruited, enrolled, and cleared of infection with a treatment dose of AL at enrolment as described above. Children enrolled in the baseline cohort will be eligible for inclusion in cohorts 1 or 2; however, those children enrolled in cohort 1 during intervention will not be eligible for inclusion in cohort 2. The recruitment, enrolment, screening, and follow-up of cohort 2 will be the same as described for the cohort 1.

Follow-up visits for each baseline, cohort 1, and cohort 2 will alternate between a clinic visit (monthly; passive case detection) and a home visit (every 2 weeks; active case detection). Routine blood sampling will occur at the clinic, whereas blood sampling will only occur during home visits if subjects have reported history of fever in the last 48 h. Study participants will be issued with an appointment card where all upcoming appointments will be recorded. In between the clinic appointments, community interviewers may refer participants to the clinic for additional assessment by the clinical officer using the participant referral slip. Participants will be instructed to visit a study health facility for any unscheduled sick visits, or they will be visited at their home if they cannot reach the clinic. Missed visits will be considered minor protocol deviations and will be collated and reported to KEMRI Scientific Ethical Review Unit (SERU) and UND along with the annual progress report. Three consecutive missed visits will prompt consideration for LTFU and withdrawal from the study.

At each visit, the parent/guardian will be asked about recent use of ITNs and other vector control interventions as well as recent history of child’s illness and recent use of anti-malarial drugs. At every clinic visit (on a monthly basis), a blood smear will be taken for malaria diagnosis. An RDT will be taken if the participant has a recent history of fever. At the intervening home visits, a blood sample will only be taken if the child has a recent history of fever or other symptoms attributable to malaria infection. Total blood volume for samples at each visit will not exceed 500 μL.

Cohort children who test positive for malaria by RDT will be treated with AL free of charge. Blood slides of children who test negative by RDT will be prioritized for examination, and treatment will be provided based on a single positive reading. However, the endpoint measurement for formal analysis will be based on examination of all blood slides by at least two expert microscopists with any discordant results resolved by a third. Cohort children will be provided a transport reimbursement for monthly scheduled clinic visits to offset the cost of coming to the clinic. Transport to hospital, if needed, will be facilitated through transport reimbursement. If there is illness or injury due to study products or procedures, fees will be paid for care at the government clinic or county or referral hospital. Referrals will be done using the general referral form. If the level of care necessary of the illness/injury is not available at the government hospital, the study will pay for care at a private health facility.

Cohorts will also be enrolled to estimate the diversionary effect of the SR intervention. Children in this cohort will be recruited from buffer zones surrounding each cluster. HHs in this area will not receive the SR or the placebo but recruitment, enrolment, screening, and follow-up procedures will be the same as described.

#### Entomological collections

Twenty clusters (10 SR, 10 placebo) will be randomly selected to estimate the impact of the SR on entomological measures of malaria transmission. Within each cluster, CDC light trap collections will be conducted in 10 randomly selected HHs within the core area of each sentinel cluster every month to assess the impact of intervention on the density of *Anopheles* mosquitoes indoors. On the same night, CDC light trap collections will be performed in 15 randomly selected HHs in the buffer zone of the same cluster to estimate the diversionary effect of the SR. The clusters for entomological sampling will be selected at the start of the baseline phase of the trial and will remain fixed throughout the study while within each selected cluster, HHs where CDC light trap collections will occur will be randomly selected each month.

In addition, quarterly HLCs will be conducted indoors and outdoors from a total of 48 HHs in a subset of 12 (6 SR and 6 placebo) of the 20 randomly selected clusters for measuring entomological endpoints. The 12 clusters will remain fixed throughout the study. Four HHs will be randomly selected in each of the clusters and will remain fixed throughout the follow-up period. Sampling will be performed for 2 weeks (6 nights per week) each quarter.

### Plans to promote participant retention and complete follow-up {18b}

Participant retention strategies include participant tracing when they miss appointments, visit reminders, calling participants after every 2 weeks for the home visit, during the follow-up phase, requests for participants to inform study staff of moves outside or within the study area, fostering relationships with participants as they engage in ancillary care at study clinics, provision of community interviewer and CO contact information for easy communication to alleviate concerns, and periodic generation of retention rates to evaluate strategies. A study SOP will be developed to provide more details on retention activities to be conducted by study staff.

### Data management {19}

#### Data forms

A combination of standardized paper-based or digital forms (programmed on Android tablets) will be used such that variable codes can be cross-referenced during interim and final analyses. KEMRI and the UND Center for Research Computing (CRC) will work together to develop the quantitative forms. Entered data (entomological and epidemiological) will be automatically assessed for quality using established quality control rules, then reviewed and appended to the data already present. Data forms can be made available upon request submitted to the UND Lead PI. All data issuing from the electronic data collection system will follow the same data collection processes outlined in the paragraphs below. Any changes to quantitative data forms will need to be agreed upon by the Site PI (in consultation with CDC technical advisors) and UND. In the event individuals cannot come to a consensus on proposed changes, the UND Lead PI will make the final decision.

#### Data quality control and quality assurance

Assigned data management study staff will be responsible for verifying data accuracy and assurances that data collection is following standardized protocols. This will promote data quality and that the trial is performed in compliance with GCP and the applicable regulatory requirements(s). Training of study staff on data collection will occur prior to commencement of baseline and throughout the trial period through refresher training events. Standardized data collection forms will be used and source data verification will occur through three primary mechanisms: (1) self-quality checks, making sure data forms are fully completed; (2) data queries, quality checks on a routine basis; (3) external monitoring, by fhiClinical, the clinical research organization responsible for trial oversight, to report on any irregularities that might be raised during monitoring visits. In addition, tablet-based digital forms will be used for data entry and uploading into the master database which will be custom designed to include rules and conditions for data variable responses (e.g., text responses cannot occur for numeric value, and thresholds for numeric data).

#### Data sharing policy

Using CommCare, KEMRI will collect data which will be securely stored on Android devices and then synchronized to the CommCare cloud. Data will then undergo an initial cleaning and de-identification by KEMRI, before being synced to UND’s central database. Data from KEMRI to UND will be transferred through a dedicated secure FTP server with password-protected access from KEMRI and CDC.

This project will generate considerable data and biological samples over the course of the 2-year study period. The data management plan will follow the guidelines and suggestions put forward by the National Institutes of Health in its online guidance document https://humansubjects.nih.gov/data_safety and by the respective institutions involved in the research (KEMRI, UND, and CDC), as well as by the community of interest (comprised of colleagues, scientists working in the same field, the biomedical community researching tropical parasitic diseases, and public health officials). The goal is transparent sharing of key findings and data so that the broad impacts of the research are meaningful and useful to key stakeholders and will therefore be shared with stakeholders as may be required.

Every consideration will be given to the nature of beneficence and justice expressed in the guiding documents for research on human subjects. Biological samples of mosquitoes and parasites will be maintained appropriately to avoid deterioration. These materials will be made available to researchers upon reasonable request and with the caveat that any forthcoming publications from research on the samples should consider the original researchers and their inclusion in the resulting publications when warranted.

#### Data storage

Any data collected on paper forms (including consent/assent forms) will be scanned and transferred to binders for storage in a secure and locked restricted access area, while all electronic captured data will be archived with a documented history of changes or corrections at the local study site.

A password-protected central study database warehousing data will be developed and managed by the UND CRC to serve as a data repository and utilized for safe data storage, extraction, integration, and analysis. The data warehouse and file repository will be backed up weekly at the local server level to ease recovery as needed.

In addition, data will be stored and backed up on the CommCare cloud. Access to study data is controlled through centralized administration, and access will be granted only with the UND Lead PI’s permission. Research records for all study subjects including history and physical findings, laboratory data, and results of consultations are to be maintained by the local Site PI in a secure storage facility at KEMRI, for a minimum of 3 years after the end of the project or until notified by grantee.

### Confidentiality {27}

Any participant information will be confidential. The results of this study will be made available to sponsors of this study but personal information will not be provided to anyone. The UND CRC will not share identifiers, but instead use a code. The code will be kept by the UND CRC, and securely at the KEMRI site according to site-specific IRB specifications and requirements for emergency situations. Raw data will be anonymized and GPS tag-blurred to remove sensitive information prior to sharing to other study sites or outside of the core study team according to local IRB requirements.

A Privacy Impact Assessment will be developed for the project, and a set of protocols and contingency plans for emergency paper-based and digital data destruction will be developed in order to guarantee privacy of research subjects in case of unforeseen risks.

### Plans for collection, laboratory evaluation, and storage of biological specimens for genetic or molecular analysis in this trial/future use {33}

This study does not include genetic or molecular analyses. Standardized protocols will be developed for the collection, storage, use, and eventual destruction of blood samples collected as part of the trial.

## Statistical methods

### Statistical methods for primary and secondary outcomes {20a}

#### Primary hypothesis

H_0_: SR does not reduce the first-time malaria hazard rate compared to placebo in Kenya.

H_1_: SR reduces the first-time malaria hazard rate compared to placebo (first-time malaria hazard ratio between SR and placebo is < 1; the expected hazard ratio is 70% or PE is 30%.

#### Secondary hypothesis

H_0_: SR does not reduce the overall malaria hazard rate compared to placebo in Kenya.

H_1_: SR reduces the overall malaria hazard rate compared to placebo (overall malaria hazard ratio between SR and placebo is < 1; the expected hazard ratio is 70% or PE is 30%).

#### Population for analysis

The intention to treat (ITT) analysis is the primary analysis approach for both the primary and secondary objectives. The ITT population includes the first recruited participant from each recruited HH that received at least one SR product or placebo according to the cluster randomization schedule. The per-protocol analysis is included as a supplementary analysis for the primary and secondary objectives. The per-protocol population includes the participants from the ITT population that are treated following the specifications of the study protocol without major protocol deviations.

#### Statistical methods—primary endpoint (ITT population)

The baseline characteristics of the enrolled subjects, HHs, and clusters will be summarized by treatment arm. Specifically, we will examine subject age and gender at the individual level; wall type and roof type, the presence of open eaves, # of windows, # of doors at the HH levels; and cluster population and baseline overall infection incidence at the cluster level.

The primary hypothesis on PE against the first-time malaria infection will be tested by comparing the hazard rates of first-time malaria infection between the SR and placebo arms upon the completion of the study in the ITT population via a cloglog model for the interval-censored time to event data. The model will include the treatment arm (SR vs. placebo), relevant baseline covariates, and a random effect that accounts for the dependency among data collected from the same cluster.

#### Statistical methods—secondary endpoints

##### PE of SR protection against the overall malaria infections

The secondary hypothesis on PE against overall new malaria infections will be tested by comparing hazard rates of the overall malaria infection between the SR and placebo arms in the ITT population using a similar approach as for the first-time infection with an additional random effect term to account for the dependency among the multiple malaria incidences collected from the same individual.

##### PE analysis without baseline covariates

A PE analysis on the first-time and the overall infections will be also performed by removing all the baseline covariates from the cloglog models and keeping “intervention group” as the only covariate (in addition to visit, as a categorical predictor per the model assumptions and setup). The hazard ratios between SR and placebo will be provided, along with 2-sided 90% CIs.

##### Diversion effect

To assess the diversion effect on the first and overall malaria infections, similar models as the cloglog models used for analyzing the primary and secondary endpoints of the first-time and overall infections will be applied, with additional covariate terms to account for the distance of each HH in the buffer zone to the boundary of the core area. The difference in the malaria first-time and overall hazard rates in the buffer zones between the SR and placebo arms at different distances to the core area will be quantified.

##### Effects of SR on entomological endpoints

The frequencies and proportions of each mosquito genus and species (anopheline and non-anophelines) collected using HLC and light trap methods will be reported by cluster and treatment arm. The time profile plots of each of aggregated entomological endpoints will be obtained over the baseline and intervention phases. An appropriate statistical model for the HBR during the intervention phase will be identified after examining the distributional characteristics of the HBR data, which will likely follow (zero-inflated) Poisson distribution or (zero-inflated) negative binomial distribution if there is over-dispersion. The model will include treatment arm (SR vs placebo), relevant baseline covariates, and a random effect that accounts for the dependency among data collected from the same cluster. The ratio between SR and placebo in HBR will be estimated. A similar model will be applied to analyze the density of mosquitoes caught by light traps. The ratio between SR and placebo on light trap density will be estimated.

The model for parity rate will be based on the (zero-inflated) Poisson distribution or a (zero-inflated) negative binomial distribution with the daily parous mosquitos as the outcome and the daily HBR as the offset and the same set of covariates as those used in the model for analyzing HBR. The model for the sporozoite rate and EIR will be similar to the parity rate. If the data on parity, sporozoite positivity, and EIR are highly unbalanced (e.g., 99% nulliparous or 99% negative for sporozoites and EIR), then the model might lead to unstable estimates or the model might not even converge. In such cases, only summary statistics will be provided.

Summary statistics will be provided on insecticide resistance at baseline and each year during the intervention phase, aggregated over the clusters on SR.

##### Relationship between epidemiological and entomological endpoints for anopheline mosquitoes

To explore the relationship between the malaria hazard rate and the entomological endpoints, similar models as the cloglog models on the overall malaria infections will be applied to the epidemiological and entomological data in the clusters from which the entomological data are collected. The outcome is the time to malaria infections with relevant baseline covariates, random effect terms to account for the dependency within the same cluster and among the multiple malaria incidences from the same individual, and additional entomological covariates (HBR and mosquito density per light traps, respectively). The regression coefficient associated with an entomological covariate quantifies the change in the malaria hazard rate on the log scale, given one unit increase in the entomological covariate.

##### Subgroup PE analysis by age group

The above analysis of the first-time and overall malaria infections in the examination of the PE and diversion effect of SR will be based on all the subjects aged 6 months to 9 years 11 months. The same set of analyses will also be performed by two age subgroups: 6 months to 59 months old, and 60 months to 9 years 11 months old to examine if the PE and diversion effects of SR differs between the two age groups.

### Interim analyses {21b}

There will be one formal interim analysis to test the primary hypothesis. The decision boundaries at the interim analysis are calculated for either stopping for futility or stopping for efficacy using the O’Brien-Fleming error spending function [36, 38–41]. Since we adopt the non-binding futility boundary [42, 43], if it is decided the study will continue due to other considerations even if we cross the futility boundary at the interim look, there will be no inflation of type I error. In other words, the trial does not need to stop to accept the null hypothesis when the test statistic falls in the futility region at the interim stage. In addition, since trials submitted to VCAG are intended to demonstrate public health value, the committee strongly recommends trials are not stopped early for benefit. In our design setting, even if the efficacy boundary is crossed at the interim look, the study may continue and there will be no inflation of type I error, as efficacy is already established at the interim. The interim analysis will occur when 528 events (50% information) are collected.

If the interim result meets the stopping criterion for futility, that is, the one-sided *p*-value for Wald’s test on the log(HR) between SR and placebo at the interim is > 0.3450, the study may stop for futility. Since we adopt the non-binding futility boundary, if it is decided the study will continue due to other considerations even if we cross the futility boundary at the interim look, there will be no inflation of type I error. If the one-sided *p*-value < 0.00882, then the study can stop for efficacy; otherwise, the study will proceed. However, if it is decided the study will continue due to other considerations even if we cross the efficacy boundary at the interim look, there will be no inflation of type I error, as efficacy is already established at the interim.

If the one-sided *p*-value at the interim is > 0.00882 but < 0.3450, then the study will continue. At the final analysis, if the one-sided *p*-value from Wald’s test on the log(HR) between SR and placebo < 0.04668, we will reject the null hypothesis, claiming SR reduces the malaria hazard rate compared to placebo in Kenya at the significance level of 5%; otherwise, we will fail to reject the null hypothesis, claiming SR does not reduce the malaria hazard rate compared to placebo in Kenya.

Interim analysis data will be available to the DSMB, Funder, Sponsor, and SCJ along with the study oversight contractor, fhiClinical, and any ad hoc experts deemed appropriate. The DSMB has the ability to recommend stopping the trial based on safety concerns, but do not have the responsibility of stopping the trial due to their assessment of efficacy or futility. The responsibility to stop the trial is held by the Sponsor.

### Methods for additional analyses (e.g., subgroup analyses) {20b}

#### Temporality of PE effects

It is expected malaria incidence changes by seasonality (rainy vs dry) and year. To examine the temporality of malaria incidence rates and the PE effect, a supplementary analysis will be performed by adding the seasonality (Jun-Dec/wet/peak and Jan-May/dry/low) and year (1 and 2) and their interaction with intervention to the covariate list in the cloglog models used for analyzing the first-time and overall infections. The PE will be estimated by seasonality and year.

#### Human behavior adjusted PE analysis

The primary and secondary analyses for the first-time infection, the overall infection, and the examination of relationship between the entomological and epidemiological endpoints will also be carried out by adjusting for the human behavior covariates the cloglog models, including “bed net usage” in the last 24 h (Y or N), “travel outside” (Y or N; an individual-level covariate), and the product application rate in each HH (expected to be close to 100%) if the data are balanced between the Y and N categories on “bed net usage” and “travel outside”, and there is practically/clinically meaningful variation in the product application rate across HHs and clusters.

#### Adjusted HBR analysis

The adjusted HBR at a given time point is calculated as the raw HBR × the proportion of people at the risk of being bitten in each HH. Specifically, in each HH where the HBR data are collected in hourly intervals from 6 pm to 6 am, the number of people indoor, the number of people outdoor, the number of people under bed net indoor, and the number of people sleeping outdoor are also collected. The adjusted HBR indoor = raw HBR × number of subjects not under the protection of bed net/ total number of indoor subject, and the adjusted HBR = raw HBR × number of subjects who sleep / total number of subject outdoor. The analysis specified for the estimating the effects SR on the raw HBR will be applied to the adjusted HBR.

#### Per-protocol analysis

If the per-protocol sample set differs from the ITT sample set, the primary analysis on the first-time infection and the secondary analysis on the overall infections will also performed in the per-protocol sample set.

### Methods in analysis to handle protocol non-adherence and any statistical methods to handle missing data {20c}

Standard Operating Procedures have been developed for all study activities, to include reporting Protocol Deviations and Protocol Violations. A Clinical Monitoring Plan has been established among the Sponsor, trial implementing partner, and the project clinical research organization—fhiClinical. fhiClinical will be responsible for managing oversight on protocol adherence through interim monitoring visits during the study phase. Departures from the protocol that are not participant-specific will be documented on a Protocol Deviation log developed by KEMRI and reported as required, and the site re-educated as necessary. Any participant-specific non-compliances and other protocol deviations will be captured in the protocol deviation Case Report Form developed by KEMRI and filed as hard copies. Major protocol deviations are to be submitted via email to applicable IRBs and the Sponsor within 24 h of the Site PI becoming aware of them, and followed by a detailed report, within 7 working days.

Significant effort will be made to avoid having missing values on outcome (malaria infection status and visit dates, and entomological endpoints). When missing values occur for an outcome for reasons not related to the outcome, reasons for missingness and the missing fraction by treatment arm and cluster will be reported. Per protocol, the subjects are screened actively on their malaria status (the outcome) every 4 weeks.

If a subject misses one or more scheduled visits due to reasons not related to the SR product or the outcome, the subject will have missing values on the outcome that can be regarded as ignorable missingness (missing at random or missing completely at random). If a subject drops out of the study due to reasons unrelated to the SR product and/or malaria infection, then the missing observations from the subject can be regarded as ignorable missingness. In both cases, all available data from the subject will be included in the primary and secondary analysis, without employing any specific technique to deal with the data, due to the ignorability of the missing mechanism.

Missing baseline covariates (individual level, household level, and cluster level) that are a part of the regression models for the outcome of interest will be imputed using simple hot-deck imputation methods if the missing fraction for the covariate is < 5%. If the missing fraction for a covariable are ≥5%, appropriate multiple imputation approaches will be applied. If a non-ignorable portion of the subjects have missing values on a covariate (due to missing at random or missing completely at random), that covariate may be excluded in the model.

### Plans to give access to the full protocol, participant-level data, and statistical code {31c}

The statistical analysis plan and analytic code will be made open access. The data and supporting information will be made available 12 months following completion of data analysis and will remain open access in the public domain.

## Oversight and monitoring

### Composition of the coordinating center and trial steering committee {5d}

UND serves are the lead organization and assumes the overall responsibility for management, oversight, and administration for the program. The coordinating personnel at UND include the Lead PI, Scientific Director, Program Manager, Program Coordinator, and Finance Manager. UND communicates on a day-to-day basis with KEMRI and CDC. KEMRI is responsible for running the cRCT on a day-to-day basis which includes but is not limited to conducting a baseline survey, deploying SRs, entomological monitoring, and subject follow-up. CDC provides technical support for the entomological and epidemiological aspects of the study. Representatives from KEMRI, CDC, and UND serve on the data management team overseeing the development and implementation of data collection, recording, and cleaning. UND will rely on fhiClinical to provide clinical oversight and monitoring of study processes, which includes but is not limited to checking enrolment, training of staff on GCP, ensuring subjects are properly consented, data are appropriately gathered, data quality, safety events are documented and reported as required, investigational product is stored, distributed and managed per specifications, and study close-out activities occur on a timely basis.

### Composition of the data monitoring committee, its role, and reporting structure {21a}

The DSMB reviews data about the safety of the Kenya cRCT and the test articles and makes recommendations about stopping the study for safety reasons. Additionally, the DSMB provides additional credibility about study quality, by reviewing regular (summary) reports from PIs during baseline and intervention phases and making recommendations as needed about study adjustment for study quality reasons. The DSMB consists of a Chair, Medical Monitor, DSMB biostatistician, and independent statistician. Members generally have no ongoing financial relationship with a trial’s commercial sponsor and will not be involved in the conduct of the trial in any role other than that of a DSMB member. Prospective members will be asked to disclose their financial relationships with any of the sponsors and/or their competitors. The DSMB reports to the Sponsor, UND. The DSMB charter can be made available upon request to UND.

### Adverse event reporting and harms {22}

The SR product contains transfluthrin, a chemical used in currently available HH mosquito control products such as mosquito coils. Exposure to the product may cause mild eye and skin irritation. These effects are usually transient and disappear after time. The product may be harmful if chewed on or swallowed, so HH owners will be advised to keep it away from children. The SR product will be fixed at a position that is out of the reach of children and it will be monitored at replacement to ensure it has not been moved. If a product is found to have been removed from its position in the HH, study staff will discuss with the HH owner to determine why it was removed and if there was any problem that led to its removal. Study staff will also reiterate safety precautions that should be taken in regard to the SR product.

AEs and SAEs of interest will be collected through passive surveillance by CHVs on children enrolled in the cohort and on other HH members who receive the study product. Additionally, AE and SAEs of interest will be solicited from study participants during follow-up visits (every 2 weeks) either in the house or clinic. We will also rely on passive surveillance of the outpatient registers at the local health facilities for reports of AE/SAE. Additionally, we may also collect unsolicited reports during compound visits and/or product replacement. Anyone experiencing the SAEs or AEs of interest will be encouraged to seek care for at the study clinic where our clinical staff will attend to them at no cost.

Unexpected SAEs affecting the cohort participants determined to be at least “possibly related” will be reported to the KEMRI SERU, study sponsor at UND, and Independent DSMB within 24 h of the PI becoming aware of the SAE. The initial report will be a short description by email, which will be followed within 7 days by a more detailed description of the SAE. AEs of interest considered at least “possibly related” will be reported to the same groups on at least an annual basis.

An AE or suspected AE is considered “serious” if it meets the following conditions:

- Results in death
- Is immediately life-threatening
- Requires in-patient hospitalization or prolongation of existing hospitalization
- Results in persistent or significant disability or incapacity
- Results in a congenital abnormality or birth defect
- Is an important medical event that may jeopardize the patient or may require medical or surgical intervention to prevent one of the outcomes listed above

All SAEs starting from enrolment until the last contact with the participant, whether or not they are related to the study product, will be reported.

AEs and SAEs during the trial will be reported in future publications. Harms will be coded in accordance with MedDRA at time of safety outcome reporting. Summary of symptom-based AE, SAE, and death reports observed during the studies will be reviewed by the trial DSMB at predetermined checks (quarterly). The AE/SAE will be labelled “Probable,” “Possible,” “Plausible,” or “Unlikely” due to SR. Summary statistics of AEs/SAEs, including mean, minimum, and maximum frequencies and percentages across clusters among enrolled subjects, will be provided by treatment arm. Statistical comparisons of the AE/SAE rates between the two arms will be conducted upon the completion of the study. Two sets of statistical analysis will be run. One set will compare the proportion of having at least one occurrence in each symptom-based AE/SAE during the whole study between the two arms, and the other will compare the total number of occurrences for each AE/SAE between the two study arms. If the data collected permits meaningful statistical hypothesis testing, *p*-values from the treatment comparisons will be reported, with multiplicity correction via the FDR approach [44].

### Frequency and plans for auditing trial conduct {23}

An independent clinical research organization, fhiClinical, will hold the responsibility of conducting clinical monitoring at the protocol implementation level, ensuring that subjects are properly consented, data are appropriately gathered, safety events are documented and reported as required, investigational product is stored, distributed and collected per specifications, and that study close-out activities occur on a timely basis.

### Plans for communicating important protocol amendments to relevant parties (e.g., trial participants, ethical committees) {25}

Protocol amendments will be submitted to the study Sponsor and local IRBs and the WHO Ethical Review Committee (ERC) for approvals. Any amendments, outcomes, analyses, or more will be communicated in person and virtually. Face-to-Face meetings will be held between Lead PI, scientific director, and site staff. Additional attendees will include the National Malaria Control Program, MOH, and other in-country public health officials. In-country meetings will also be convened with civil society members, religious leaders, and key beneficiaries. These meetings will be critical as the trial progresses and the topics addressed will be pertinent to further execution of the trial. Due to the project design and geographic location of study team, teleconferences will be another method of formal project communication. Stakeholder teleconferences will be scheduled as needed to review and assess study progress and issues. Teleconference agendas will be drafted and distributed by UND.

### Dissemination plans {31a}

The plan for dissemination of results includes submission to WHO/VCAG, workshop with study partners, on-site meetings in Kenya, and presentations at scientific meetings and/or peer-reviewed publications.

## Discussion

ITNs and indoor residual spraying have contributed to substantial reductions in malaria burden since 2000 [1]. However, malaria remains a serious public health concern, particularly in sub-Saharan Africa due to gaps in protection from ITNs and IRS. Supplemental tools such as SRs may address these gaps in coverage and contribute to further reductions in malaria burden [5, 7, 45].

SRs, like the Mosquito Shield™, are devices containing volatile chemicals that disperse in air under ambient conditions (no requirement of electricity or heat to volatilize); they can be placed inside or around houses. The volatile chemicals introduced into the air repel mosquitoes from entering the treated space and/or disrupt mosquito biting and feeding habits, possibly impacting their survival and reproductive behavior [5]. SR products are envisioned to complement and enhance existing vector control methods due to the continual release of volatile AI which precludes the requirement of mosquito contact with a treated surface.

The role for SR products in malaria public health vector control are likely to be greatest in settings where early-evening and/or outdoor biting by *Anopheles* mosquitoes avoids the effects of ITNs and IRS. In addition, SR product chemicals operate through a different mode of action and laboratory assays have demonstrated behavioral effects against both insecticide susceptible and resistant anopheline and mosquito vectors responsible for transmitting multiple human pathogens [27]. Thus, SRs may serve as a tool in areas where insecticide resistance limits the effectiveness of ITNs and IRS. SRs may also reduce selection pressure for insecticide resistance and thereby maintaining the tools’ natural life span [7].

There are thousands of registered SR products already on the market and used for protection from nuisance biting. Current registered products include sophisticated, expensive products used in the USA and Europe (liquid vaporizers) as well as inexpensive and simpler products (e.g., mosquito coils) that are widely used throughout Africa and Asia. However, there is presently no public sector use of mosquito coils (or any other SR product format) for disease control due to insufficient evidence for WHO policy recommendation.

Over the past decade, formal national and international meetings have been convened to bring together academics, industry, funders, and global public health experts, including representatives from the WHO, to discuss the role of SR products in the reduction of arthropod-borne diseases. A critical aspect of these meetings and subsequent efforts has been to establish a critical path of development for SR products based on expert advice. This includes measures related to scientific, regulatory, and social parameters. In part, these criteria outline the endpoints of a target product profile (i.e., optimum product characteristics) for SR products. A SR vector control product class is currently under WHO assessment for public health value. However, the biggest evidence gap at the moment is the lack of sufficient epidemiological data needed to demonstrate public health impact across a range of eco-epidemiological settings to inform a potential WHO policy recommendation for the incorporation of SR products into current disease control programs. In 2017, the WHO VCAG recommended additional clinical trials to evaluate SR against malaria in Africa [46]. These knowledge gaps must be addressed to inform WHO SR policy recommendation. Once/if the WHO VCAG endorses a policy recommendation for the SR class to be recommended for public health use, national disease control programs will have the option of adopting a SR policy and “next-in-kind” SRs (e.g., with active ingredients to include other volatile pyrethroids such as metofluthrin) will have the opportunity to be marketed within the public health channel without the need to undergo WHO VCAG assessment, incentivizing SR product research.

This trial will generate evidence to support decision-making by WHO to recommend SR products for public health use and inform considerations of a national policy to include SRs in vector-borne disease control programs. Outputs will align with those of other global health stakeholders addressing residual malaria transmission, insecticide resistance, and access and barriers to market introduction of new vector control products. If the SR product is effective, it may be deployed in malarious areas to complement other vector control interventions such as ITNs to help mitigate the problems of insecticide resistance and/or vector biting at time where ITNs may be ineffective to further drive malaria towards elimination.

## Trial status

Under protocol version 7 from November 20, 2020, recruitment for the baseline cohort began March 1, 2021, and final subject enrolment was completed April 2, 2021.

The study has currently completed the baseline phase (as of July 31, 2021), whereby participants have been recruited, screened, and enrolled and followed up without intervention for 4 months. Baseline data analyses are ongoing to verify underlying assumptions of malaria incidence, coefficient of variation, and loss to follow-up. Recruitment, screening, and enrolment of subjects for follow-up with intervention is scheduled to commence in August 2021.

## Supplementary Information

**Additional file 1.** Statistical analysis plan

**Additional file 2.** WHO Trial Registration Data Set

## Abbreviations

AE: Adverse events
AEGIS: Advancing Evidence for the Global Implementation of Spatial Repellents
AI: Active ingredient
AL: Artemether-Lumefantrine
CAB: Community Advisory Board
CDC: Centers for Disease Control and Prevention
CHV: Community health volunteers
CI: Confidence interval
CRC: Center for Research Computing
cRCT: Cluster-randomized control trial
CV: Coefficient of variance
DSMB: Data Safety and Monitoring Board
EIR: Entomological inoculation rate
ERC: Ethical Review Committee
HBR: Human biting rate
HH: Household
HLC: Human landing catch
ICF: Informed consent form
IRS: Indoor residual spraying
ITN: Insecticide-treated net
ITT: Intention to treat
KEMRI: Kenya Medical Research Institute
LLIN: Long-lasting insecticidal net
LTFU: Loss to follow-up
MOH: Ministry of Health
PE: Protective efficacy
PI: Principal Investigator
RDT: Rapid diagnostic test
SAE: Serious adverse event
SCJ: SC Johnson, A Family Company
SERU: Scientific Ethical Review Unit
SR: Spatial repellent
UND: University of Notre Dame
VCAG: Vector Control Advisory Group
WHO: World Health Organization
