# Supplementary material for: Evaluation of the protective efficacy of a spatial repellent to reduce malaria incidence in children in western Kenya compared to placebo: study protocol for a cluster-randomized double-blinded control trial (the AEGIS program)
Source: Trials. 2022 Apr 5;23:260. doi: 10.1186/s13063-022-06196-x (PMC8980512; doi:10.1186/s13063-022-06196-x)
Supplement: Supplementary file 1 — Additional file 1. Statistical analysis plan [file 13063_2022_6196_MOESM1_ESM.pdf]

Spatial Repellent Products for Control of Vector Borne Diseases

Kenya

Statistical Analysis Plan

Version 6.2

Nov 11, 2021

Prepared by Fang Liu

fang.liu.131@nd.edu

Summary of changes of the Kenya Study SAP

Version	Date	Summary of Major Changes
V1.0	May, 2018	A very early draft
V2.0	June 18, 2018	Revised based on the feedback from UNITAID consortium meeting on May 30 and 31, 2018
V3.0	Aug 14, 2018	Added more details to the secondary and tertiary objectives and the associated endpoints.
V4.0	Sep 11, 2018	Corrected the terminology in the description of the entomological endpoints
V4.1	Oct 19, 2018	study design changed from one cohort with 12-month follow-up t one cohort with 18-month follow-up
V4.2	Dec 19, 2018	study design changed from one cohort with 18-month follow-up t two cohort with 12-month follow-up each
V4.3	Apr 14, 2019	Proofreading before sending to VCAG for review
V4.4	July 21, 2019	Addressed VCAG off-cycle comments
V4.4.1 V4.4.2	Aug 27, 2019	Added the adjusted HBR analysis based on the feedback from the JHU
V4.4.3	Nov 7, 2019	Replotted Figure 1 to clarify the study design schematic
V5.0	Feb 13, 2020	Revised according to the Nov 12, 2019 VCAG comments
V5.1	Nov 18, 2020	Reduced the upper age limit at time of enrollment from 15 years to 9 years and 11 months, per the request of WHO ethical panel to remove pregnancy testing from the study procedures.
V5.2	Jan 19, 2021	Corrected a typo ("overall new Pf malaria infection" was misplaced under "primary endpoint" in Sec 3 and should be under "secondary endpoints" in Sec 3)
V5.3	March 4, 2021	Modified the subsection (7.4) on how missing data will be handled; adjusted the definition of new infection and protection period after malaria clearance treatment in Sec 6.
V6	Aug 26, 2021	Adjustment of sample size based on the results from the baseline data analysis. Specifically, one cluster (#43) had 0 malaria infections during the 4-month baseline period. If included in the intervention phase, the data from the cluster could bias the PE estimation. This version of the SAP is based on the scenario of dropping cluster #43 and one additional cluster to maintain the balance between treatment arms (29 clusters per arm instead of the originally planned 30). We reduce the study power from the original 85% to 80% to mitigate the impact on the change in the # of households per cluster from dropping two clusters. There are no other changes in the SAP.
V6.1	Sep 2, 2021	Added the justification for removing clusters #43 and #9 in Section 4 (Study Design) and corrected a few typos.
V6.2	Nov 1, 2021	1) Revised the safety and AE analysis plan per the request of the UNITAID DSMB; 2) added the formula for calculating EIR

Contents

Summary of changes of the Kenya Study SAP 2
1 Objectives 4
2 Hypotheses 4
3 Endpoints 4
4 Study Design 5
5 Population for analysis 6
6 Definition of new malaria infection 7
7 Statistical Methods 8
7.1 Primary endpoint (ITT Population) 8
7.2 Secondary endpoints (ITT Population) 9
7.3 Supplementary analysis 12
7.4 Handling of missing data 13
7.5 Analysis of baseline 14
7.6 Interim analysis 14
8 Software 14
9 Sample Size Determination 14
Reference 15
Appendix 16
I. Sample size under different assumptions of baseline incidence rate and between-cluster CV 16
II. Mock Tables and Figures 17
III. Some sample SAS and R procedures used in the analysis 21

1 Objectives

Primary Objective

1. To evaluate the protective efficacy (PE) of spatial repellent (SR) against the first-time malaria infection in the core zones in Kenya.

Secondary Objectives

1. To evaluate the PE of SR against all malaria infections (both first-time and recurrent) in the core zones.
2. To evaluate the PE of SR against first-time and overall malaria infections by two age subgroups (≤ 59 months old; 5 years old to 9 years and 11 months old) in the core zones.
3. To assess the diversion effect (dispersion effect) of SR on malaria infections (both first-time and overall) in Kenya.
4. To evaluate the effect of SR on anopheline-human contact using human biting rate (HBR) as an indicator indoor and outdoor for all anopheline and by anopheline species.
5. To evaluate the effect of SR on anopheline survival and population age structure using parity rate as an indicator for all anopheline and by anopheline species.
6. To evaluate the effect of SR on anopheline infectivity using sporozoite rate as an indicator for all anopheline and by anopheline species.
7. To evaluate the effect of SR on anopheline infectivity using entomological inoculation rate (EIR) as an indicator for all anopheline and by anopheline species.
8. To evaluate the effect of SR on light trap indoor density for each anopheline species and for all anopheline in both the core zone and the buffer zone
9. To investigate the relationships between the reduction in first-time and overall malaria infection and the HLC HBR, mosquito density by light trap indoor density, the parity rate, the sporozoite rate, and the EIR for all anopheline and by anopheline species.
10. To evaluate SR insecticide resistance at baseline and each year during the intervention period and to explore its relationship with PE
11. To assess the safety of SR.

2 Hypotheses

Primary Hypothesis

H_0: SR does not reduce the first-time malaria hazard rate compared to placebo in Kenya.

H_1: SR reduces the first-time malaria hazard rate compared to placebo (overall malaria hazard ratio between SR and placebo is < 1; the expected PE is 30%)

Secondary Hypothesis

H_0: SR does not reduce the overall malaria hazard rate compared to placebo in Kenya.

H_1: SR reduces the overall malaria hazard rate compared to placebo (overall malaria hazard ratio between SR and placebo is < 1; the expected PE is 30%)

3 Endpoints

The primary endpoint is the first-time malaria infection in the core zones from the intervention follow-up period. The second endpoints include:

1. Overall new malaria infection during the intervention follow-up period in the core zones.

2. The first-time and overall malaria infections during the follow-up period in the buffer zones.
3. The first-time and overall malaria infections by two age groups (≤ 59 months old; 5 years old to 9 years and 11 months old)).
4. Anopheline-human contact using HBR as an indicator indoor and outdoor for all anopheline and by anopheline species.
5. Anopheline survival and population age structure using parity rate as an indicator for all anopheline and by anopheline species.
6. Anopheline infectivity using sporozoite rate as an indicator for all anopheline and by anopheline species.
7. Anopheline infectivity using EIR as an indicator for all anopheline and by anopheline species.
8. Light trap indoor density for each anopheline species and for all anopheline in both the core zone and the buffer zone.
9. Baseline and annual insecticide resistance.
10. Adverse events and serious adverse events.

4 Study Design

The original study design had 30 clusters per treatment arm during both the baseline and intervention periods. The analysis results of the baseline data (read 1 of microscopy) on Aug 9 suggested that **Cluster 43** has zero malaria infections during a 4-momth baseline period. For this reason, we have proposed to VCAG, and received endorsement, to remove Cluster 43 (the zero-incidence cluster) and one additional study cluster (two clusters total) in order to maintain balance between treatment arms. The resulting ratio of SR to placebo is 29:29 (58 total clusters) vs. 30:30 originally proposed.

Cluster 09 is the second cluster selected to be removed from the intervention phase. This cluster was selected by consensus from the UND, KEMRI and CDC teams based on the following criteria: 1) Cluster 09 was excluded from the entomology sampling frame during baseline due to the rapid urbanization of the village, therefore baseline entomology endpoints will not be affected; 2) Cluster 09 is experiencing rapid urbanization trends similar to Cluster 43 which will lead to challenges in SR product application inside homes due to housing construction (cemented painted walls as opposed to mud walls), as more people are refusing installation of the wall hanging devices(hooks, screws and tapes) as well as potential bias in exposure to malaria risk. Cluster 09 also happened to have the third-lowest baseline incidence rate based on the preliminary baseline data analysis.

The study design is a cluster randomized trial with 29 clusters per intervention arm (SR and Placebo), with a 4-month baseline follow-up (60 clusters in total), one month of baseline data evaluation before randomization, and a total of post-randomization follow-up period of 24 months. The clusters post randomization will be split into two temporally and spatially non-overlapping cohorts, and each cohort will be followed up for 12 months after they receive intervention.

For the evaluation of the primary objective, 28 households are recruited from each cluster or equivalently 14 households per cohort per cluster (factoring in a 35% loss-to-follow-up rate). For the evaluation of the secondary objective on the diversion effect, 40 households or equivalently 20 households per cohort are recruited from each cluster (factoring in a 35% loss-to-follow-up rate). One kid aged from 6 months old to 9 years and 11 months old from each household is recruited

for the biweekly (every 2 weeks) malaria check-up during the intervention follow-up period. The design schematic is illustrated in Figure 1 below.

Twenty clusters (10 SR, 10 placebo) will be randomly selected to estimate the impact of the SR on entomological measures of malaria transmission. Within each cluster, light trap collections will be conducted in 10 randomly selected households within the core area of each sentinel clusters every month to assess the impact of SRs on the density of Anopheles mosquitoes indoors. On the same night, light trap collections will be collected in 15 randomly selected households in the buffer area of the same cluster to estimate the diversion effect of the spatial repellent. The indoor/outdoor human landing catches (HLC) will be conducted in 4 houses in each of 12 clusters (6 intervention and 6 control) every quarter.

**Baseline Cohort** (4-month follow-up + 1-month evaluation)	**Intervention Cohorts**	
	**12 months (Cohort 1)**	**12 months (Cohort 2)**
	**Core zone** (29clusters on SR and 29 clusters on PBO with 28 HHs per cluster)	
60 clusters;<br>20 HHs per cluster	SR (29 clusters; 14 HHs per cluster)	
	PBO (29 clusters; 14 HHs per cluster)	
		SR (29 clusters; 14 HHs per cluster)
		PBO (29 clusters; 14 HHs per cluster)
	**Buffer zone** (29 clusters on SR and 29 clusters on PBO with 40 HHs per cluster)	
60 clusters;<br>14 HHs per cluster	SR (29 clusters; 20 HHs per cluster)	
	PBO (29 clusters; 20 HHs per cluster)	
		SR (29 clusters; 20 HHs per cluster)
		PBO (29 clusters; 20 HHs per cluster)

Figure 1: Study Design Schematic for PE evaluation

5 Population for analysis

The intention to treat (ITT) analysis is the primary analysis approach for both the primary and secondary objectives. The ITT population includes the first recruited kid from each recruited household that receives at least one SR product or placebo per the cluster randomization schedule. If a recruited subject comes from a household used for entomological data collection, that subject will be not used in the ITT analysis. The per-protocol (PP) analysis is included as a supplementary analysis for the primary and secondary objectives. The PP population includes the subjects from the ITT population that are treated following the specifications of the study protocol without major protocol deviations.

5.1 Subjects who moves to a new house during the intervention follow-up period

- For a subject who moves to a different house within the same cluster, that subject will be included in both the ITT and PP analyses. The household characteristics will be updated at the time the subjects moved.
- For a subject who moves to a different house in a different cluster, the data from the subject before the subject moves will be included in the ITT analysis. All data from the subject will be included in the PP analysis, both the treatment information and the household characteristics will be updated at the time the subjects moved.

5.2 Subjects who are hospitalized for serious complicated illness (e.g. chronic illness), die, drop out, or miss scheduled visits due to reasons not related to the malaria outcome or intervention during the follow-up period

For subjects that fall under this category, the available data from the subjects (up to the time point when the subjects are hospitalized, die, or drop out; data from the scheduled visits that the subjects did not miss) will be included in both the ITT and PP analyses as the missing or absent data can be ignored (see Section 6.4 of the SAP for more details).

5.3 Subjects who do not receive (complete) intervention due to travelling outside, mis-application or partial application of the product

For the ITT analyses, these subjects will be included as is. For the PP analysis, “travel outside” (Y or N; an individual-level covariate) and the product application rate in each household (expected to be close to 100%) will be included as covariates if the data are not overly imbalanced between the Y and N categories for “travel outside”, and there is practically/clinically meaningful variation in the product application rate across households and clusters.

6 Definition of new malaria infection

Following a positive malaria diagnosis, whether the next positive malaria diagnosis, either during the active or passive screening periods, is a new infection or just a remnant or a carryover from the previous malaria infection depends on the time lapse between the two malaria infections, and whether and when the first malaria infection is treated.

The treatment for malaria infection last for 3 days. Denote the first day of treatment by Day 1. A diagnosis test of malaria will take place on Day 15 +/- 3 days (that is, Day 12 to Day 18). If a positive malaria infection is detected during the diagnosis test, then the malaria infection will not be regarded as a new infection but rather a carryover and a second round of 3-day treatment will be conducted. The number of subjects that are infected but will not treated is expected to be minimal (to be confirmed at interim and final analyses of the baseline data). If there is no treatment for an infection, the subsequent positive will be regarded as a new infection only with one negative blood slide between the two positives.

The positive diagnosis that cannot be treated as a new infection will be re-coded as negative before any the following statistical analysis on malaria infection (baseline, first-time infection, overall malaria infection) is applied. Correspondingly, for the overall incidence rate calculation, the time at the risk will be adjusted by subtracting the “protection period” (the number of days from Day 1 treating a malaria infection and the post-treatment diagnosis test on Day 15 +/- 3 days).

Also noted is that the active screening of malaria occurs every 4 weeks and the passive screening occurs in between two active screenings only when a subject experiences a fever. It is possible there are only a few passive screenings upon the completion of data, leading to data imbalance between the odd-numbered visits (the active screening) and the even-numbered visits (the passive screening). To deal with this problem if it occurs, we will apply the following approach. If the passive screening in a visit is negative on malaria, then that data point will be removed as it contains no additional info on malaria or time at risk on top of the active screenings before and after it. If the passive screening is positive, then the passive positive will be assigned to either the active screening visit immediately before the passive screening or after, whichever is closer to the passive screening in time. The same approach will be applied to the analysis of first-time overall malaria infections in Sec 7.1 and 7.2.

7 Statistical Methods

The statistical analysis and results reporting will follow the CONSORT guidelines for CRTs[1].

7.1 Primary endpoint (ITT Population)

The baseline characteristics of the enrolled subjects, households, and clusters will be summarized by treatment arm. Specifically, we will examine subject age and gender at the individual level, wall type and roof type, floor height, house open eaves, # of window, # of doors at the household levels, and cluster population and baseline overall infection incidence at the cluster level.

The primary hypothesis on PE against the first-time malaria infection will be estimated by comparing the hazard rates of first-time malaria infection between SR and placebo upon the completion of the study in the ITT population. The complementary-log-log (cloglog) regression model $\log(-\log(1-\theta_{kit})) = \beta_{0t} + \boldsymbol{x}_{ki}^T\beta_1 + z_k$ will be applied [2-7]. The cloglog model is a proportional hazard model for interval-censored data. θ_{kit} is the discrete time hazard rate of subject i in cluster k at time t, and $\boldsymbol{x}_{ki}$ contains the individual-level (age, gender), household-level (number of doors, number of windows, open eaves Y or N, wall type, roof type), and cluster-level (baseline incidence rate, cluster population size, intervention group) covariates. If the data are extremely unbalanced in a categorical covariate (not in terms of the distributions between the treatment arms, but the marginal distribution of the variable itself; e.g., 99% households have the same type of walls vs 1% do not) or if a non-ignorable portion of the subjects have missing values on a covariate (due to MAR or MCAR), that covariate may be excluded in the model. $z_k \sim N(0, \sigma_1^2)$ is the random effect at the cluster level. The null hypothesis of PE = 0% is equivalent to $\beta = 0$, which will be tested by the Wald's test $z = \hat{\beta}/s$, where s is the estimated standard error of $\hat{\beta}$. PE is estimated by $(1-\exp(\hat{\beta})) \times 100\%$, where $\hat{\beta}$ is the estimated regression coefficient associated with the treatment group, and $\exp(\hat{\beta})$ is the estimated hazard ratio (HR) between SR and placebo, with a 95% upper-sided CI (the hypothesis is one-sided).

There will be one formal interim analysis to test the primary hypothesis and make decision on whether stopping the trial or not. The decision boundaries are calculated for either stopping for futility or stop for efficacy using the O'Brien-Fleming error spending function [8-10]. Since we adopt the non-binding futility boundary, if it is decided the study will continue due to other considerations even if we cross the futility boundary at the interim look, there will be no inflation of type I error. In other words, the trial does not need to stop to accept the null hypothesis when the test statistic falls in the futility region at the interim stage. In addition, since trials submitted to VCAG are intended to demonstrate public health value, the committee strongly recommends that trials are not stopped early for benefit. In our design setting, even the efficacy boundary is crossed at the interim look, the study may continue and there will be no inflation of type I error, as efficacy is already established at the interim.

The interim analysis will occur when 528 events (50% information) are collected. Assuming a baseline incidence rate of 3.0 per person-year and constant average hazard rates for cohort 1 and cohort 2 post intervention, the interim analysis will occur around the end of the intervention follow-up on Cohort 1 (i.e., the end of Month 12 post randomization). The decision boundary for futility and efficacy is presented in Figure 2.

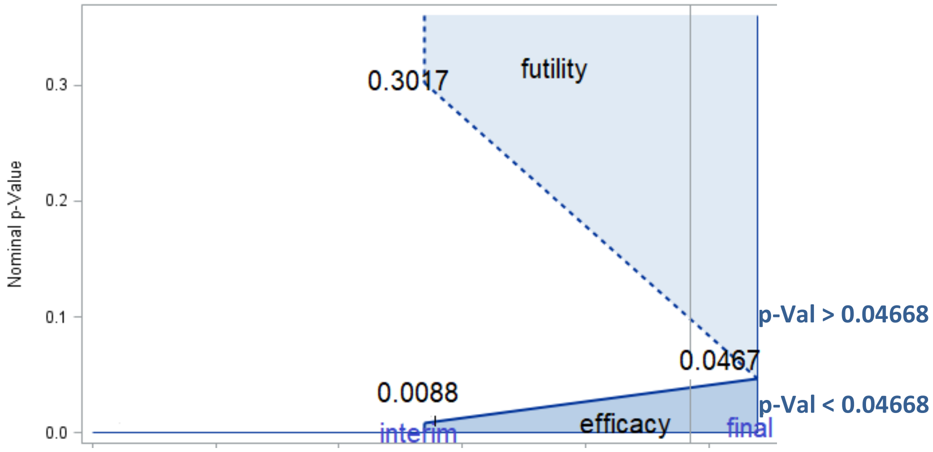

Figure 2: Boundary plot (in p-value) for testing the primary hypothesis on PE (1-sided Type-I error rate α = 0.05, Type-II error rate β = 0.2; between-cluster CV = 47.4%, baseline incidence rate = 3 per person-year; O'Brien-Fleming error spending function)

If the result meets the stopping criterion for futility, that is, the one-sided p-value for the Wald's test on the log(HR) between SR and placebo at the interim look > 0.3017 (see Figure 2), then the study can stop for futility. Since we adopt the non-binding futility boundary, if it is decided the study will continue due to other considerations even if we cross the futility boundary at the interim look, there will be no inflation of type I error. In other words, the trial does not need to stop to accept the null hypothesis when the test statistic falls in the futility region at the interim stage. If the one-sided p-value < 0.00882, then the study can stop for efficacy; otherwise, the study will proceed. However, if it is decided that the study will continue due to other considerations even if the efficacy boundary is crossed at the interim look, there will be no inflation of type I error, as efficacy is already established at the interim.

If the one-sided p-value at the interim is > 0.00882 but <0.3017, then the study continues. At the final look, if the one-sided p-value from the Wald's test on the log(HR) between SR and placebo < 0.0467, we reject the null hypothesis, claiming SR reduces the malaria hazard rate compared to placebo in Kenya at the significance level of 5%; otherwise, we fail to reject the null hypothesis, claiming SR does not reduce the malaria hazard rate compared to placebo in Kenya.

7.2 Secondary endpoints (ITT Population)

No interim analysis will be performed on any of the secondary endpoints listed below.

PE and diversion effect of SR protection again the first-time malaria infection

The secondary hypothesis on PE against the overall new malaria infections, as defined in Sec 6, will be tested by comparing the hazard rates of the overall malaria infection between SR and placebo in the ITT population. The cloglog regression model $\log(-\log(1-\theta_{kit})) = \beta_{0t} + \boldsymbol{x}_{ki}^T\beta_1 + z_k + z_{i(k)}$ will be applied. θ_{kit} is the discrete time hazard rate of subject i from household j in cluster k at time t, and $\boldsymbol{x}_{ki}$ contains the individual-level (age, gender), household-level (number of doors, number of windows, open eaves Y or N, wall type, roof type), and cluster-level (baseline incidence rate, cluster population size, intervention group) covariates. If the data are extremely unbalanced in a categorical covariate (not in terms of the distributions between the treatment arms, but the marginal distribution of the variable itself; e.g., 99% households had the same type of walls vs 1% that did not) or if an non-ignorable portion of the subjects have missing values on a covariate

(due to MAR or MCAR), that covariate maybe may be excluded in the model. $z_k \sim N(0, \sigma_1^2)$ and $z_{i(k)} \sim N(0, \sigma_2^2)$ are the random effects at the cluster, household, and individual levels respectively. The cloglog model is a proportional hazard model and thus HR does not depend on time *t*. The null hypothesis of PE = 0% is equivalent to $\beta = 0$, which will be tested by the Wald's test $z = \hat{\beta}/s$, where s is the estimated standard error of $\hat{\beta}$. A one-sided p-value and 95% upper-sided confidence interval (CI) for PE will be provided (the hypothesis is one-sided). If the one-sided p-value between SR and placebo < 0.05, we reject the null hypothesis, claiming SR reduces the overall malaria hazard rate compared to placebo in Kenya at the significance level of 5%; otherwise, we fail to reject the null hypothesis, claiming SR does not reduce the overall malaria hazard rate compared to placebo in Kenya.

<u>Diversion effect</u>

To assess the diversion effect on the first and overall malaria infection a similar model as the model used for analyzing the primary endpoint in Sec 7.1 and 7.2 will be applied. The only difference in the diversion effect assessment is the inclusion of the distance of each household in the buffer zone to the nearest intervention house in the core zone, and the interaction term between the distance and treatment arm in addition to the covariates listed in the model in Sec 7.1 and 7.2. How the malaria first-time and overall hazard rates compare in the core zones of SR relative to those of the placebo at different distance to the core zone will be quantified.

<u>Subgroup PE analysis by age group</u>

The above analysis of the first time and overall malaria infections in the examination of the PE and diversion effect of SR will be based on all the subjects aged 6 months to 9 years and 11 months old. The same set of analysis will also be performed by two age subgroups (≤ 59 months old; 5 years old to 9 years and 11 months old) to examine if PE and diversion effects of SR will differ by two age groups.

<u>PE analysis without baseline covariates</u>

A PE analysis on the first-time and the overall infections will be also performed by removing all the baseline covariates from the cloglog models presented Sec 7.1 and 7.2 and only keeping "intervention group" as the only covariate (in addition to visit, as a categorical predictor per the model assumptions and set-up). The hazard ratios between SR and placebo will be provided, along with 2-sided 90% CIs.

<u>Incidence rate estimation and mid-point imputation of time to infection.</u>

The first-time and overall malaria incidence rates per person-year during the whole intervention follow-up will be calculated by cluster for the SR and the placebo arms respectively. The first-time malaria incidence rate is defined as the ratio of the number of first-time malaria cases during the whole study vs sum of the time to event/time at risk (in year) across the individuals within the same cluster, and the overall malaria incidence rate is defined as the ratio of the number of new malaria cases during the whole study vs sum of the time to event/time at risk (in year) for each of the new cases across the individuals within the same cluster.

Since the active screenings of malaria incidences are either every 4 weeks (active screening) with passive screening taken between two active screenings if fever is reported, the actual time for contracting malaria is unknown (interval censored). Therefore, the mid-point between two consecutive screenings will be used as the time at risk for a malaria event that occurs in the latter screening. The average per-person-year first-time and overall malaria incidence rates in the SR

and the placebo arms, and the incidence ratios between the two will be calculated, together with the coefficients of variation in both arms on both incidence endpoints.

With the mid-point imputed time to infection, we will also run Cox regression with the same list of covariates as in the cloglog models for the 1st-time and overall malaria infections to estimate the hazard ratios between SR and placebo. The cloglog models are based on fixed time intervals for interval censored data (every two weeks +/-3 days in this study) for each subject. If an ignorable portion of samples in the collected data deviate scheduled visit, Cox regression models will be applied to estimate the PE against the 1st-time and overall malaria infections on the mid-point imputed time to infection data.

Effects SR on entomology

The endpoints in the entomological analysis include the HBR (number of anopheline- caught during the 12-hr interval overnight), anopheline parity rate, anopheline sporozoite rate, and anopheline EIR, and the anopheline indoor density collected by light-trap.

We will report the frequencies and proportions of each mosquito Genus and species (anopheline and non-anophelines) collected using HLC and light trap methods across clusters and treatment arm. In addition, the following analysis will be performed for each anopheline species and for all anophelines.

The time profile plots of each of aggregated entomological endpoints will be obtained over the baseline and intervention period. An appropriate statistical model for the HBR will be identified after examining the distributional characteristics of the HBR data, which are likely to follow (zero-inflated) Poisson distribution, or (zero-inflated) negative binomial distribution if there is over-dispersion. The covariates in the models will include the fixed effects of intervention, time, cluster population size, number of houses in a cluster, location (inside or outside), location and intervention interaction; and a random effect for cluster. The ratio between SR and placebo in HBR will be estimated, and the %change in HBR by SR is given by (1-HBR ratio)×100%.

The model for parity rate will be the (zero-inflated) Poisson distribution or a (zero-inflated) negative binomial distribution with the daily porous mosquitos as the outcome and the daily HBR as the offset, and the same set of covariates as those used in the model for analyzing HBR. The model for the sporozoite rate will be similar to the parity rate with the change of outcome variable to daily mosquitos with positive sporozoite. Note that if the data on parity and sporozoite positivity are highly unbalanced (not in terms of the distributions between the treatment arms, but the marginal distribution of the variable itself; e.g., 99% nulliparous or 99% negative sporozoite), then the model might lead to unstable estimates or the model might not even converge. In such cases, only summary statistics will be provided.

The time profile plots of each of light trap indoor density will be obtained over the baseline and intervention period. An appropriate statistical model will be identified after examining the distributional characteristics of the light trap indoor density which are likely to follow (zero-inflated) Poisson distribution, or (zero-inflated) negative binomial distribution if there is over-dispersion. The covariates in the models will include the fixed effects of intervention, time, cluster population size, and a random effect for cluster. The ratio between SR and placebo on light trap indoor density will be estimated.

EIR is defined as the number of infective mosquito bites a person receives per unit time (typically annually) and is calculated as

$$\text{HBR} \times \text{sporozoite rate} = \frac{\text{\# of mosquitos collected}}{\text{\# of capture nights}} \times \frac{\text{\# of sporozoite positive mosquitos}}{\text{\# of mosquitos tested}}.$$

We will calculate the EIR per household per cluster per unit time and provide summary statistics at baseline and per year during the intervention period by treatment group.

Summary statistics will also be provided for insecticide resistance during the intervention period by treatment.

Analysis of the relationship between malaria hazard rate and entomological endpoints

To explore the relationship between the malaria hazard rate and the entomological endpoints, a similar model as the cloglog models used to address the primary objective on the first-time malaria infection and the secondary endpoint on the overall malaria infection will be applied to the epidemiological and entomological data in the clusters from which the entomological data are collected. The random effects and the individual-level and, household-level covariates will be similar to the cloglog models used for the PE analysis. The cluster-level covariates in the model will include the baseline incidence rate, cluster population size, and a covariate that captures the entomological information. Specifically, for HBR, the measurement to be paired with a malaria diagnosis in an individual is average daily HBR taken within 7 to 28 days before the diagnosis over the two-week period and over the sentinel households where entomological endpoints are collected in the same cluster to which the individual belongs. The regression coefficient associated with log(HBR) quantifies the change in the malaria hazard rate on the log scale, given one unit increase in log(HBR). For parity rate and sporozoite positivity rate, as long as there is enough data collected on these two endpoints and they are not highly unbalanced with regard to its marginal distribution (e.g., 99% mosquitos caught are nulliparous or sporozoite negative), the relationship between the malaria hazard rate and those two will also be investigated in a similar fashion as for HBR.

Safety assessment

Summary of symptom-based adverse events (AE), severe adverse events (SAE), and death reports observed during the studies will be reviewed by the trial DSMB at predetermined checks (quarterly). The AE/SAE will be labelled "Probable", "Possible", "Plausible", "Unlikely" due to SR. Summary about AE/SAE, including mean, minimum and maximum frequencies and percentages across clusters among enrolled subjects, will be provided by the treatment arm. Statistical comparisons of the AE/SAE rates between the two arms will be conducted upon the completion of the study. Two sets of statistical analysis will be run. One set will compare the proportion of having at least one occurrence in each symptom-based AE/SAE during the whole study between the two arms, and the other will compare the total number of occurrences for each AE/SAE between the two study arms. If the data collected permits meaningful statistical hypothesis testing, p-values from the treatment comparisons will be reported, with multiplicity correction via the FDR approach [11].

7.3 Supplementary analysis

Temporality of PE effects

It is expected malaria incidence changes by seasonality (rainy vs dry) and year. To examine the temporality of malaria incidence rates and the PE effect, a supplementary analysis will performed by adding the seasonality (Jun-Dec/wet/peak) and Jan-May/dry/low) and year (1 and 2) and their interaction with intervention to the covariate list in the cloglog models used for analyzing the first-time and overall infections. The PE will be estimated by seasonality and year.

Human behavior adjusted PE analysis

The primary and secondary analyses laid out in Sections 7.1 and 7.2 for the first-time infection, the overall infection, and the examination of relationship between the ento- and epi- endpoints will also be carried out by adjusting for the human behavior covariates the cloglog models, including "bednet usage" in the last 24 hrs (Y or N), "travel outside" (Y or N; an individual-level covariate), and the product application rate in each household (expected to be close to 100%) if the data are balanced between the Y and N categories on "bednet usage" and "travel outside", and there is practically/clinically meaningful variation in the product application rate across households and clusters.

Adjusted HBR analysis

The adjusted HBR at a given time point is calculated as the raw HBR× the proportion of people at the risk of being bitten in each household. Specifically, in each household where the HBR data are collected in the hourly interval from 6pm to 6am, the number of people indoor, the number of people outdoor, the number of people under bednet indoor, the number of people sleeping outdoor are also collected. The adjusted HBR indoor = raw HBR × number of subjects not under the protection of bednet/ total number of indoor subject, and the adjusted HBR = raw HBR × number of subjects who sleep / total number of subject outdoor. The analysis specified for the estimating the effects SR on the raw HBR in Sec 7.2 will be applied to the adjusted HBR.

Per-protocol analysis

If the PP sample set differs from the ITT sample set, per the criteria listed in Sec 5, the primary analysis on the first-time infection and the secondary analysis on the overall infections as listed in Sec 7.1 and 7.2 will also performed in the PP sample set.

7.4 Handling of missing data

Significant effort will be made to avoid having missing values on outcome (malaria infection status and visit dates, and entomological endpoints). When missing values occur for an outcome for reasons not related to the outcome, reasons for missingness and the missing fraction by treatment arm and cluster will be reported. Per protocol, the subjects are screened actively on their malaria status (the outcome) every four weeks.

- If a subject misses one or more scheduled visits due to reasons not related to the SR product or the outcome, the subject will have missing values on the outcome that can be regarded as ignorable missingness [12] (MAR or MCAR).
- If a subject drops out study due to reasons unrelated to the SR product and/or malaria infection, then the missing observations from the subject can be regarded as ignorable missingness[11] (MAR or MCAR).

In both cases, all the available data from the subject will be included in the primary and secondary analysis, without employing any specific missing data analysis techniques, due to the ignorability of the missing mechanisms [11].

Missing baseline covariates (individual-level, household-level, and cluster-level) that are a part of the regression models for the outcome of interest will be imputed using simple hot-deck imputation methods if the missing fraction for the covariate is <5%. If the missing fraction for a covariable are ⩾5%, appropriate multiple imputation approaches will be applied. If a non-ignorable portion of the subjects have missing values on a covariate (due to missing at random or missing completely at random), that covariate may be excluded in the model.

7.5 Analysis of baseline

The per-person-year first-time and overall malaria incidence rate from the 58 recruited clusters will be calculated. Since the malaria incidences are collected on a biweekly basis, the mid-point between two visits will be imputed as the time at risk for a malaria event. The average incidence rate will be calculated, together with the coefficients of variation. The baseline analysis will occur at Month 2 and Month 4 during the baseline period internally on blinded data.

7.6 Interim analysis

A formal interim analysis during the intervention period will be to examine the primary hypothesis as outlined in Section 6.1 of this SAP. The interim analysis will be performed by an independent DSMB who will recommend whether to continue or stop a trial. The final decision to stop should always rest with the DSMC, not the investigators, or the funder.

The baseline data will be analyzed at the mid-point of the baseline period.

No other interim analyses are planned.

8 Software

Software used will be SAS for Windows, Version 9.4 or higher (SAS Institute, Cary, NC, USA) and Rstudio Version 1.0.143 or higher (RStudio, Inc, Boston, MA, USA).

9 Sample Size Determination

The sample sizes below might be adjusted based on the data collected during the 4-month baseline period. Since the adjustment will only utilize the baseline data (baseline incidence and CV) with intervention information, the Type-I error rate will not be inflated.

Primary hypothesis on first-time malaria infection

The sample size determination on the required number of households per cluster for testing the primary hypothesis on PE is based on the hazard rate comparison in the proportional hazards regression model [13-14]. With the following specifications: 1-sided type-I error rate = 5% (because the primary hypothesis is one-sided as SR is unlikely to increase the hazard rate of malaria infection compared to placebo), true PE = 30%, a between-cluster coefficient of variation (CV) of hazard rate = 48% (based on the historical data collected from Kenya), one interim analysis for efficacy and non-binding futility, with the O'Brien-Fleming error spending function when 50% information is collected, then 1056 independent malaria events will need to be observed to reach 80% power in testing the primary hypothesis on PE.

With a baseline first-time malaria infection hazard rate of 3.0 per person-year (ppy), 29 clusters per treatment, 28 households per cluster, factoring in a 35% loss to follow-up (LTFU) rate, are expected to yield 1056 independent first-time malaria events within 12 months follow-up period per cohort post randomization to yield 80% power. The 28 households will be split in half between the two sequential cohorts with 14 households per cohort per cluster. If, by the end of the 2-year study, 1056 independent malaria events are not reached, the study may extend until 1056 events are collected without inflating the type I error rate in the testing of the primary hypothesis.,

Appendix I provides the sample size requirement under different assumptions of baseline incidence rate (ppy) (3, 2, and 1.5) and between-cluster CV (%) (44%, 50% and 60%).

Secondary hypothesis on overall malaria infection

The sample size calculated to yield 80% power for establishing the primary hypothesis on first-time infection PE is expected to lead to 80% power when it comes to testing the secondary hypothesis on the overall malaria infection. This is because that the baseline overall malaria incidence rate is likely to be similar 3.0 per person-year (the assumed first-time incidence rate) and with a similar CV value (both are confirmed by the baseline data); in addition, there is no interim analysis on the second hypothesis and all the pre-specified Type-I error will be used toward testing the PE against overall infection upon the completion of the study.

Quantification of the diversion effect

Since there is no formal hypothesis on the diversion effect objective on the diversion effect is estimation, we focus on determining a practical feasible SS that gives a relative high precision for the estimated PE of SR in the near-zone on the malaria incidence rate. With a 48% between-cluster CV, and a baseline incidence rate of 3.0 per person year, 29 clusters with 30 HHs per cluster lead to a half width of 0.278 for the 95% CI on the log-scale, that is, the ratio between the upper bound of the 95% CI vs. the point estimate is 1.320 for a hazard ratio estimate between SR and placebo. Factoring in a 35% LTFU rate, the required sample size is 40 households in the buffer zone per cluster. The total households will be split in half between two sequential cohorts, with 20 households per cohort per cluster.

Note: Since the sample sizes for both PE and diversion effect evaluations already factors in the LTFU rate, there is no need for replacement subjects unless the LTFU is larger than assumed. If replacement subjects are to be recruited, they should not have been exposed to the intervention until the time they are considered for replacement.

Reference

1. Marion K Campbell, Gilda Piaggio, Diana R Elbourne, Douglas G Altman (2012), Consort 2010 statement: extension to cluster randomised trials, BMJ 345:e5661
2. J. D. Kalbfleisch, R. L. Prentice (2002, 2nd ed), The Statistical Analysis of Failure Time Data, Wiley-Interscience
3. P. D. Allison (1982), Discrete-Time Methods for the Analysis of Event Histories, Sociological Methodology, 13: 61-98
4. D. Collett (2002, 2nd ed), Modelling Binary Data, Second Edition, Chapman & Hall
5. B. P. Carlin and J. S. Hodges (1999), Hierarchical proportional hazards regression models for highly stratified data, Biometrics.55(4):1162-1170
6. V. Henschell, J. Engel, D. Hölzel and U. Mansmannina (2009), A semiparametric Bayesian proportional hazards model for interval censored data with frailty effects, BMC Medical Research Methodology, 9:9
7. G. Yin and J. G. Ibrahim (2005), A Class of Bayesian Shared Gamma Frailty Models with Multivariate Failure Time Data, Biometrics, 61(1):208-216
8. Eighth VCAG meeting report (Section Conditions for early termination of trials) https://apps.who.int/iris/bitstream/handle/10665/273106/WHO-CDS-VCAG-2018.01-eng.pdf?ua=1
9. Bretz F, Koenig F, Brannath W, Glimm E, Posch M. (2009), Adaptive designs for confirmatory clinical trials. Stat Med. 28(8):1181–217.

10. Schüler S, Kieser, M, Rauch G (2017), Choice of futility boundaries for group sequential designs with two endpoints BMC Med Res Methodology. 17: 119.

11. Benjamini Y, Hochberg Y (1995). *Controlling the false discovery rate: a practical and powerful approach to multiple testing*, Journal of the Royal Statistical Society, Series B. **57** (1): 289–300

12. R. J. A. Little and D. B. Rubin (2002), Statistical Analysis with Missing Data (2nd edition), J. Wiley & Sons, New York.

13. M. Lievens, J. J. Aponte, J. Williamson, B. Mmbando, A. Mohamed, P. Bejon and A. Leach (2011), Statistical methodology for the evaluation of vaccine efficacy in a phase III multi-centre trial of the RTS,S/AS01 malaria vaccine in African children, Malaria Journal, 10:222

14. The RTS,S Clinical Trials Partnership (2011), First Results of Phase 3 Trial of RTS,S/AS01 Malaria Vaccine in African Children, The New England Journal of Medicine, 365(20): 1863-1875.

Appendix

I. Sample size under different assumptions of baseline incidence rate and between-cluster CV

The table below shows, for different between-cluster coefficient of variation (CV) of hazard rate and different baseline incidence rate, that the required number of events and the number households per cluster (factoring in a 35% LTFU rate) for establishing the primary hypothesis on PE with 85% power with 30 clusters per arm under the 1-sided type-I error rate = 5% with true PE = 30% and one interim analysis for efficacy and non-binding futility with the O'Brien-Fleming error spending function when 50% information is collected.

Note the table was provided since the first draft of SAP to show how sample size changes under different assumptions on the parameters that affects the power/sample size calculation. The table is not updated after the dropping two clusters based on the baseline data analysis, as the trend in sample size calculations would be similar for 30 vs 29 clusters per arm. The power/sample size is updated in the main text in Sec 9 with 29 clusters per arm.

	Baseline incidence (ppy)	between-cluster CV		
		44%	50%	60%
# clusters per arm		30	30	40
# events total *		637	1467	2739
# HHs per cluster	3	16	36	51
	2	18	41	58
	1.5	21	47	65

II. Mock Tables and Figures

Figure A1: flow diagram of progress of clusters and individuals

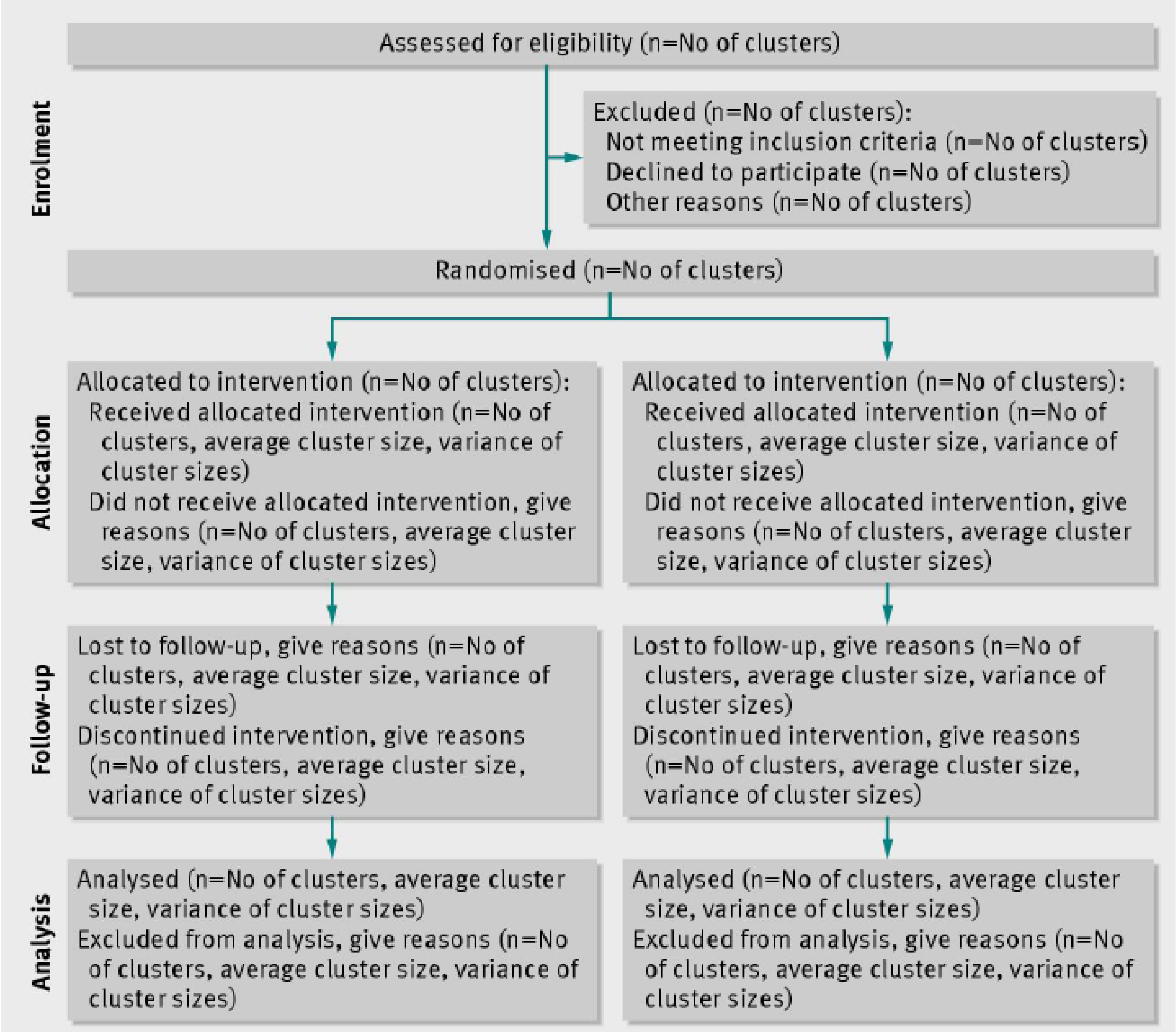

From Campbell et al. (2012): *Consort 2010 statement: extension to cluster randomized trials*) [1]

Table A1: Summary on baseline covariates

**Individual level**		
	**SR**	**Placebo**
Age in months (mean ± SD, (min, max))<br>Gender (% of boys)		
**Household level**		
	**SR**	**Placebo**
house wall type (%, n)<br>house roof type (%, n)<br>Floor height (mean ± SD, n)<br>house open eaves (%, n)<br># of windows (mean± SD, n)<br># of doors (mean± SD, n)		
**Cluster level**		
	**SR**	**Placebo**
Cluster population (mean ± SD, (min, max))		
Baseline overall infection incidence per person-year (mean ± SD, (min, max))		

Table A2: Protective Efficacy (PE) of SR against all infections

Look	Treatment	Baseline incidence rate	# of households	# of infections	Time at risk (in years)	hazard ratio (95% CI)	PE (95% CI)
1	SR						
	placebo						
2	SR						
	placebo						

Baseline coefficient of variation (CV) of incidence rate: xxx%

Similar tables will be provided on the 1) 1st-time malaria infections, 2) by-age group analysis on PE; and 3) the supplementary PE analysis for 1st-time, overall, and by-age group infections with no covariates included in the cloglog model.

Table A3: Diversion effect of SR

Distance from the core zone	Treatment	Baseline incidence rate	# of households	# of events	Time at risk (in years)	hazard ratio (95% CI)	PE (95% CI)
d_1	SR						
	placebo						
….	SR						
	placebo						
d_K	SR						
	placebo						
	Similar tables will be provided for 1st-time malaria infection, and for overall malaria						

Table A4: Effects of SR compared to placebo on the entomological endpoints

	Mean (95% CI)		Ratio (95% CI)
Endpoint	SR	Placebo	SR vs. placebo
HBR			
Parity rate			
sporozoite positivity rate			
Indoor density (light trap)			

Figure A2: Kaplan Meier Curves for SR and placebo on 1st-time malaria infections

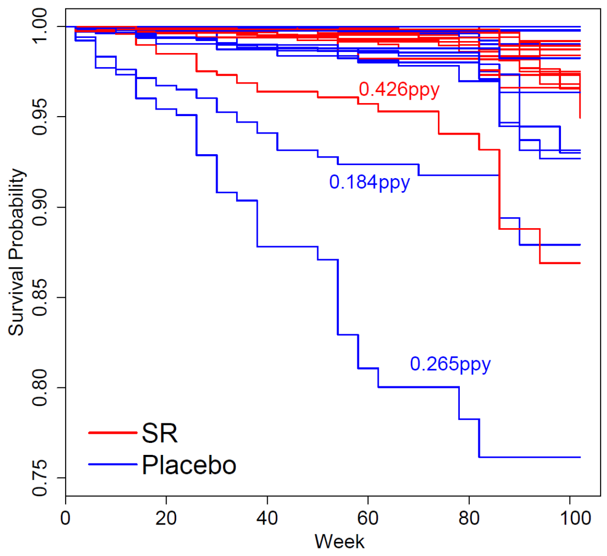

The KM curves will be generated for all recruited subjects, and also by age group (≤ 59 months old; 5 years old to 9 years and 11 months old).

Figure A3: time profile of estimated HBR (mean +SD)

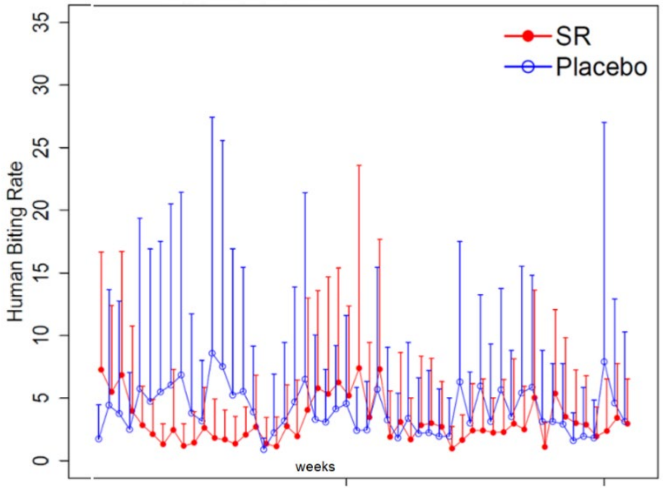

Table A5: AE and SAE Summary Statistics

	Mean (Min, Max) Frequency (Percentage) across Cluster		
	SR related*	SR	Placebo
Adverse Event/Symptoms **			
Skin irritation/Rash	Possible		
Runny nose	Possible		
Nausea/Vomiting	Possible		
Salivation/Drooling	Plausible		
Cough	Unlikely		
Eye irritation	Unlikely		
Headache	Unlikely		
Diarrhea	Unlikely		
Abdominal discomfort	Unlikely		
Difficulty Breathing	Unlikely		
Other	Unlikely		
Serious Adverse Event **			
Congenital anomaly/ Birth defect	Unlikely		
Death	Unlikely		
Persistent or Significant Disability or Incapacity	Unlikely		
Hospitalization or prolongation of existing hospitalization	Unlikely		
Other	Unlikely		
* Defined per SJC safety tests.<br>** AE and SAE summaries of the clinical diagnosis (disease) may be added			

Table A6: Statistical Comparison on having at least AE and SAE

	Frequency (percentage)		SR vs. placebo (p-Value)	
AE	SR (N= xxx)	Placebo (N= xxx)	raw	adjusted*
Skin irritation/Rash				
Runny nose				
Nausea/Vomiting				
…..				
* FDR-adjusted p-value				

Table A7: Statistical Comparison on the total number of AE and SAE

	Frequency		SR vs. placebo (p-Value)	
AE	SR (N= xxx)	Placebo (N= xxx)	raw	adjusted*
Skin irritation/Rash				
Runny nose				
Nausea/Vomiting				
…..				
* FDR-adjusted p-value				

III. Some sample SAS and R procedures used in the analysis

Note the final codes for KM curves and estimation if PE could differ slightly from the sample codes below, which are meant to demonstrate the main procedures/commands in R and SAS to run those two types of analyses rather than to be followed strictly.

a) KM curves for each cluster. Some sample codes are given below.

```
library(interval)
fit<-icfit(Surv(left,right,type="interval2")~treatment, data=malaria)
plot(fit)
```

b) For estimating the PE of SR against first-time and overall malaria infection
SAS procedure `PROC glimmix` with the `cloglog` link. Each subject will have multiple rows, one for each visit. The statement `random` will be included to take account of the dependency of the subjects from the same cluster. Some sample codes are given below.

```
proc glimmix data=WORK.IMPORT NOCLPRINT method=LAPLACE;
class  Subject_ID  Cluster  Final_Diagnosis  Treatment_Allocation  Gender
Eaves_Open wallwood recoded_visit;
model Final_Diagnosis (event='POS')=
Treatment_Allocation
Gender
Eaves_Open
wallwood
age_scaled
Number_of_Doors
clusterpop_scaled
BaselineIncidence
recoded_visit/ dist=binary solution link=cloglog;
random int / subject=Cluster;
estimate 'Treatment' Treatment_Allocation 1 -1 /alpha=0.1 cl exp;
ods output estimates=treatest; run;
```
